# The Medial Septum Is Insulin Resistant in the AD Presymptomatic Phase: Rescue by Nerve Growth Factor-Driven IRS_1_ Activation

**DOI:** 10.1007/s12035-018-1038-4

**Published:** 2018-05-07

**Authors:** Valentina Sposato, Nadia Canu, Elena Fico, Salvatore Fusco, Giulia Bolasco, Maria Teresa Ciotti, Matteo Spinelli, Delio Mercanti, Claudio Grassi, Viviana Triaca, Pietro Calissano

**Affiliations:** 1 European Brain Research Institute (EBRI) Rita Levi-Montalcini Foundation, Viale Regina Elena 295, Rome, Italy; 2 National Research Council (CNR), Institute of Cell Biology and Neurobiology, Via del Fosso di Fiorano 64, Rome, Italy; 3 Department of System Medicine, Section of Physiology, University of Rome “TorVergata”, Rome, Italy; 4 Department of Biotechnological and Applied Clinical Sciences, University of L’Aquila, L’Aquila, Italy; 5 Institute of Human Physiology, Università Cattolica del Sacro Cuore, Rome, Italy; 6 European Molecular Biology Laboratory (EMBL), Monterotondo Outstation, Rome, Italy; 7 Fondazione Policlinico Universitario Agostino Gemelli, Rome, Italy

**Keywords:** Basal forebrain cholinergic neurons, Brain insulin resistance, Alzheimer’s disease, NGF, IRS1

## Abstract

Basal forebrain cholinergic neurons (BFCN) are key modulators of learning and memory and are high energy-demanding neurons. Impaired neuronal metabolism and reduced insulin signaling, known as insulin resistance, has been reported in the early phase of Alzheimer’s disease (AD), which has been suggested to be “Type 3 Diabetes.” We hypothesized that BFCN may develop insulin resistance and their consequent failure represents one of the earliest event in AD. We found that a condition reminiscent of insulin resistance occurs in the medial septum of 3 months old 3×Tg-AD mice, reported to develop typical AD histopathology and cognitive deficits in adulthood. Further, we obtained insulin resistant BFCN by culturing them with high insulin concentrations*.* By means of these paradigms, we observed that nerve growth factor (NGF) reduces insulin resistance in vitro and in vivo. NGF activates the insulin receptor substrate 1 (IRS_1_) and rescues c-Fos expression and glucose metabolism. This effect involves binding of activated IRS_1_ to the NGF receptor TrkA, and is lost in presence of the specific IRS inhibitor NT157. Overall, our findings indicate that, in a well-established animal model of AD, the medial septum develops insulin resistance several months before it is detectable in the neocortex and hippocampus. Remarkably, NGF counteracts molecular alterations downstream of insulin-resistant receptor and its nasal administration restores insulin signaling in 3×Tg-AD mice by TrkA/IRS_1_ activation. The cross-talk between NGF and insulin pathways downstream the insulin receptor suggests novel potential therapeutic targets to slow cognitive decline in AD and diabetes-related brain insulin resistance.

**Electronic supplementary material:**

The online version of this article (10.1007/s12035-018-1038-4) contains supplementary material, which is available to authorized users.

## Introduction

The basal forebrain cholinergic system (BFCS) modulates many important behaviors, through robust inputs to the cortex and hippocampus [1, 2] and reciprocal feedback projections [3]. In particular, the cholinergic neurons of the medial septum, corresponding to the Ch1 subregion of the BFCS, modulate attention, reference and spatial memories, as well as learning in mice and humans [1, 4]. Early synaptic failure and metabolic derangement in basal forebrain cholinergic neurons (BFCN) have been reported to parallel memory deficits in mild cognitive impairment (MCI) and in Alzheimer’s disease (AD) [5–7]. For this reason, it has been proposed as a good predictor of MCI progression toward AD [8]. BFCN are considered an energy-demanding neuronal population because of their dependency upon acetyl-CoA for biosynthesis of both ATP and acetylcholine. In line with this, their high metabolic profile has been suggested to be the main cause of peculiar BFCN vulnerability in neurodegenerative diseases [9].

Neuronal metabolism and plasticity, and higher cognitive functions are regulated by the insulin pathway in the central nervous system [10, 11]. Insulin is both produced locally in the brain [12] and transported from the circulation, concentrating in the cortex and forebrain tissues. Insulin receptors (IR) and Insulin receptor substrates (IRS) are widely distributed in the mammalian forebrain [10, 13, 14]. In particular, IRS_1_ is the most studied isoform involved in the control of glucose homeostasis and brain physiopathology [15]. The docking of IRS to the IR upon insulin binding initiates a survival cascade mediated by PI3K/AKT and CREB, leading to elevation of c-Fos expression, translocation of the glucose transporters (Glut) to the plasma membrane [16, 17], and consequent glucose uptake, boosting the neuronal activity [18]. On the other hand, neuronal metabolic impairment caused by blunted or deficient insulin signaling, called insulin resistance, affects neuronal functions and has been suggested to parallel the onset and progression of AD pathology [19, 20]. High IRS_1_ phosphorylation at inhibitory serine residues is a general consensus marker of brain insulin resistance [20]. Aberrant IRS_1_ phosphorylation at S312, S616 and/or S636 (equivalent to rodent S307, S612 and S632, respectively) mainly exerted by GSK3beta (feedback control) and by JNK1/2 ad PKC zeta/lambda (feed-forward inhibition) [21, 22] is the result of a chronic maladaptive mechanism that attenuates insulin signaling. Elevated serine phosphorylated IRS_1_ level has been reported in the brain of a primate AD model as well as in AD patients, and is associated with early synaptic dysfunctions and late stage neurodegeneration in AD [23].

Of interest for our study, insulin has been demonstrated to regulate the expression of choline acetyltransferase (ChAT), the key acetylcholine (Ach) biosynthetic enzyme, in SH-SY5Y neurons [24] and in the retina [25]. Indeed, also other insulin pathway activators, like IGFI, IGFII and PPAR agonists, stimulate ChAT levels and sustain survival and differentiation of cholinergic neurons [26, 27]. Accordingly, perturbed insulin signaling is associated with swelling of cholinergic neurites and reduced ChAT immunoreactivity in the medial septum of 3×Tg-AD mice, a common model of AD [28, 29]. Impaired acetylcholine homeostasis has been also described in streptozotocin (STZ)-treated rats, a rodent model of diabetes-related insulin resistance [30]. Taken together, all these experimental observations strongly indicate a cause-effect relationship between insulin resistance and cholinergic damage. Moreover, the insulin control of the cholinergic phenotype implicates that insulin pathway perturbations may contribute to cholinergic transmission deficits in AD. Hence, to ascertain whether BFCN develop insulin resistance in the AD brain is essential to understand the early molecular events underlying the AD pathology. While central insulin resistance has been recently observed in the neocortex and in the hippocampal region in 3×Tg-AD and Tg2576 mice [31], its potential impact on the BFCS remains unexplored. 

To investigate the insulin pathway in healthy and AD affected BFCN, we nasally administered insulin to wild-type and 3×Tg-AD mice, and analyzed the activation of the insulin signaling in the medial septum, a BFCN enriched region of the BFCS. Our study shows that the classic insulin pathway is elicited by nasal insulin administration in the medial septum of wild-type (wt) mice but not of age and sex-matched 3×Tg-AD mice, suggesting a condition reminiscent of insulin resistance. Noteworthy, we set up and fully characterized an in vitro model of insulin resistance developed in cholinergic neurons, to study the underlying molecular events and assess candidate drug, like nerve growth factor (NGF), for counteracting the insulin-resistant state of BFCN. 

This cellular model may be of interest for the design of novel therapeutic strategies in AD and brain-related metabolic disorders. It enabled us to show that NGF not only elicits the insulin pathway and controls glucose metabolism by Glut2 translocation to the plasmamembrane in healthy BFCN, but it also reduces BFCN insulin resistance by re-activating the IRS_1_-driven insulin pathway in vitro and in AD mice.

## Methods

### Rodent Strains

Triple transgenic AD (3xTg-AD) mice, harboring human APP Swedish, presenilin M146V and tauP301L mutations [32], and C57 Bl6/J mice were purchased (Jackson Lab), and housed in the Animal Facility of the Università Cattolica Medical School. Purchased Wistar rats (HARLAN Laboratories Ltd., Füllinsdorf, Switzerland) were housed at the Animal Facility of the “Institute of Cellular Biology and Neurobiology”, National Research Council (IBCN-CNR; Rome, Italy) at the European Center of Brain Research (CERC). Animals were handled in compliance with the National (D.Lgs26/2014) and European Union legislation guidelines for animal welfare (2010/63/EU). All efforts were made to minimize the number of animals used and suffering.

### Reagents and Antibodies

Murine NGF was purified from submaxillary glands [33]. Bovine insulin (I2643; SIGMA, St. Louis, MO, USA) and the specific IRS inhibitor tyrphostin NT157 (Axon Medchem BV; Groningen, The Netherlands) were purchased. NGF from Xiamen Bioway (Biotech Co., Ltd., China) was also used in the study.

Primary antibodies: anti-Trk (SANTA CRUZ, Santa Cruz, CA, USA, sc7268), anti-ChAT (MILLIPORE, Temecula, CA, USA, AB144P), anti pIR (tyr1150/1151) (CELL SIGNALING, Danvers, MA, USA, 3024; ABCAM, Cambridge, UK, ab5500), anti-IR (CELL SIGNALING, Danvers, MA, USA, 3024; MILLIPORE, Temecula, CA, USA, 05–1104), anti-pY IRS_1_ (Tyr608) (MILLIPORE, Temecula, CA, USA, 09–432; ABCAM, Cambridge, UK, ab66153), anti-pS IRS_1_ (Ser307; clone 24.6.2, MILLIPORE,Temecula, CA, USA, 05–1087), anti-IRS_1_ (Biorbyt), anti-IRS_1_ IHC-plus (LIFESPAN BIOSCIENCES Inc., Seattle, WA, USA, LS-B1373), anti-pAKT (Ser473) (CELL SIGNALING, Danvers, MA, USA, 4051), anti-AKT (11E7) (CELL SIGNALING, Danvers, MA, USA, 4685), anti-pJNK1/2 (Thr183/Tyr185) (CELL SIGNALING, Danvers, MA, USA, 9251), anti-JNK (CELL SIGNALING, Danvers, MA, USA, 9252), anti-pGSK3β (Ser9; CELL SIGNALING, Danvers, MA, USA, 9323), anti-GSK3β (27C10) (CELL SIGNALING, Danvers, MA, USA, 9315), anti-Glut2 (ABCAM, Cambridge, UK, ab54460), and anti-GLUT4 (ABCAM, Cambridge, UK, ab654). Secondary antibodies: anti mouse-HRP and anti-rabbit-HRP (PerkinElmer, Waltham, MA, USA); donkey anti mouse-546, donkey anti rabbit-488, and donkey anti goat-647 (Life Technologies, Carlsbad, CA, USA).

### Nasal Insulin Administration

The procedure involves the immobilization of the mouse and the direct administration of small volumes of the selected substances (2.5 μl per nostril) using a Gilson type P10 precision pipette. Awake mice were gripped by the skin of their necks and held gently, but firmly, upside-down in the palm of the hand. The tip is placed near the nostril so that the animal can inhale the drug directly from the tip. The operator makes a slight pressure on the pipette piston in order to facilitate the expulsion of the drug. The procedure is extremely rapid (about 1 min) and painless, and it does not require any anesthesia [34]. Mice were intranasal administered with bovine insulin (0.125 IU) or vehicle (saline) and sacrificed after 20′ or 40′, to assess the optimal treatment duration in order to achieve the activation of the medial septum. The septum was surgically removed from the brain and collected for further analysis. The activation of the insulin pathway (IR-IRS-AKT) was analyzed by western blotting (Suppl. Fig. 1 a-d) and showed that while IR and IRS1 were phosphorylated already after 20′ and are still elevated after 40′ insulin treatment, AKT phosphorylation required a longer treatment (40′). Based on these results, the duration of the nasal treatment was set at an intermediate time point (30′) allowing the concomitant detection of all the molecules of interest. For the rescue experiment, vehicle, NGF (40 μg), or insulin (0.125 IU) were nasally administered to 3×Tg-AD mice and, after 30 min, mice were sacrificed. Brain tissues were removed and frozen until further biochemical analysis. Mice were starved for 14–16 h before intranasal stimulation.

### Primary Cholinergic Neurons

Cholinergic neurons were harvested from E17 Wistar rat embryos, as previously described [35, 36]. Dissociated cells were plated in Neurobasal medium supplemented with 2% B27 (Invitrogen Inc., Carlsbad, CA, USA) for 10 days (37 °C, 5% CO_2_) and then used for experiments (DIV10). To obtain insulin resistant cholinergic neurons, DIV10 neurons were daily incubated with insulin (2μM) for 72 h (DIV13) and then used for experiments. Cholinergic neurons were first washed three times with medium, then starved (90′), and treated with insulin (10 nM, 30′) or NGF (100 ng/ml, 30′). Starvation by replacement of culture medium with Neurobasal Medium without B27 avoids confounding effects from B27-derived insulin and insulin-related serum factors. Cells were seeded as follows: 1.5 × 10^6^ cells on poly-l-lysine (SIGMA) coated 35 mm plates (BD Falcon, Durham, NC, USA; 353001) for western blotting analyses and 5 × 10^4^ cells on glass coverslips in 24-wells plates (BD Falcon; 351147) for immunofluorescence analyses and glucose uptake assay.

### Western Blotting

Tissues samples and cultured neurons were digested in a RIPA buffer with “complete protease and phosphatase inhibitory cocktail” (Roche) and centrifuged (10,000 rpm, 20′). The supernatants were collected, and the amount of total protein was determined by Quick Start Bradford Dye Reagent. Each sample (40 μg) was separated by SDS-PAGE in precast 4–12% Bis-Tris Plus gels (Bolt, Invitrogen), transferred to nitrocellulose membranes (0.45 μM, GE Healthcare), and incubated for 1 h at room temperature with 10% non-fat dry milk in TBS-T (10 mM Tris, pH 7.5, 100 mM NaCl, and 0.1% Tween-20). The overnight incubation with primary antibody (4 °C) was followed by incubation with the appropriate HRP-conjugated secondary antibody (1:2000, Pierce, 1 h, RT) and the ECL substrate (32106; Pierce- Thermo Scientific). The films were digitized using a professional scanner (HP Scanjet 4050) and quantified by gel densitometry using the ImageJ software (NIH). Measurements were standardized between the experimental groups using the same calibration system and a fixed threshold over the background. Data are expressed as percentage optical density relative to control group, and presented as means ± SEM.

### Immunofluorescence Labeling and Microscopy

Primary cultures were fixed for 20 min in PBS containing 4% paraformaldehyde, permeabilised with PBS plus 0.3% Tween, and quenched by ammonium chloride (50 mM, 30′, RT). Aspecific staining by the secondary antibody was blocked by incubation with normal donkey serum (10%, 1 h, RT). The overnight incubation (4 °C) with primary antibodies was followed by the appropriate secondary antibodies (1:2000, 1 h, RT).

Triple immunofluorescence with rabbit anti-IR (1:100), mouse anti-IRS_1_ (1:70), and goat anti-ChAT (1:200) antibodies was performed, followed by incubation with a mixture of donkey anti rabbit-Alexa488, anti-mouse Alexa546 and anti-goat Alexa647 secondary antibodies. Rabbit anti-Glut2 (1:200) or rabbit anti-Glut4 (1:500) followed by donkey anti-rabbit Alexa488 were used to characterize glucose metabolism. Moreover, mouse anti-Trk (1:100) plus rabbit anti-pIRS_1_ (1:100) or mouse anti-Trk (1:100) plus rabbit anti-IRS_1_ (1:70) antibodies followed by a cocktail of donkey anti-mouse Alexa543 and donkey anti-rabbit Alexa488 were used for double immunostaining, as control for primary antibody specificity in the PLA experiment. Neurons were also counterstained with 4′,6-diamidino-2-phenylindole (DAPI; Life Technologies), mounted on coverslips with Prolong Gold Antifade Mounting (Life Technologies) and kept at − 20 °C before image analysis. Confocal microscopy was performed with the laser scanning confocal microscope TCS SP5 (Leica Microsystems, Mannheim, Germany) using a 40X (NA = 1.25) and a 63X (NA = 1.4) oil-immersion lens. A UV Diode laser operating at 405 nm, an Argon laser at 488 nm, a HeNe laser at 543 nm were used as excitation sources. Representative images were chosen among 30 immunostained neurons from at least 3 different experiments.

### Nuclear c-Fos Staining and Analysis

c-Fos staining was performed by overnight incubation of control and insulin resistant cholinergic neurons with rabbit anti-c-Fos antibody (1:500), followed by secondary anti-rabbit Alexa Fluor-488 antibody (1:2000, 1 h, RT). Nuclei were counterstained by DAPI (1:1000; 15 min, RT). c-Fos immunofluorescence was acquired with an epifluorescent microscope (Leica CTR5500; Leica Microsystems) equipped with a CCD camera (Leica). Images for direct comparison were collected using the same settings. The number of c-Fos positive over the total number of DAPI stained nuclei per field (field area = 0.366 μm^2^; ×20 objective) was measured in 5 different fields per coverslip, in at least three coverslips per experimental group. The analysis was performed after calibrating for particle pixels size (50–400 pixels) and applying a fixed threshold over the background. Nuclei were counted both by manual and automated counting methods (ImageJ software, NIH) with comparable results. Data are expressed as percentage to control group (CTR) and presented as mean ± sem.

### Glucose Uptake Assay

Primary cholinergic neurons were seeded at 2-5x10^4^cells/well on a glass coverslips. Neurons were starved (90′) by incubation with Neurobasal medium without B27 supplement (37 °C, 5% CO_2_). The glucose uptake measurement was performed by means of the “GluTracker Glucose Uptake cell-based Kit” following manufacturer’s instructions (K681; Biovision Inc.). The bright green fluorescence generated by the fluorescent 2-deoxy-glucose conjugate (GluTracker) is proportional to the amount of glucose taken up by cells and can be used as a direct measure of the fluorescent glucose analogue (GluTracker) uptake. Briefly, the GluTracker mix was prepared as follow and added to each well: 376 μl of neurobasal medium, 4 μl GluTracker Reagent, and 20 μl of GluTracker Enhancer. Cholinergic neurons were incubated at 37 °C with 5% CO_2_ for 30 min. After incubation, the cells were washed once with ice-cold 1X Analysis Buffer (500 μl), and then replaced with fresh 1X Analysis Buffer (200 μl). Then, neurons were fixed for 20 min in PBS containing 4% paraformaldehyde, counterstained with DAPI (Life Technologies), and mounted on coverslips with Prolong Gold (Life Technologies). Images were acquired with an epifluorescent microscope (Leica CTR5500; Leica Microsystems) using a blue excitation fluorescence filter (excitation range: 420-495 nm) and a × 20 objective. The total immunofluorescence intensity per field (field area = 0.366 μm^2^) was measured in 5 different fields per coverslips, in at least 4 coverslips per experimental group. Data reported in the graphs are expressed as percentage relative to control group and presented as mean ± sem.

### In Situ Proximity Ligation Assay (PLA)

The interaction of TrkA with pIRS_1_^Y608^ and IRS_1_ were detected by PLA assay, a sensitive technique allowing detection of direct binding (<40 nM distance) between endogenous proteins [37]. Specific primary antibodies (working dilution: 1:100) and species-specific secondary antibodies (PLA probes), each conjugated to a unique short DNA strand (MINUS and PLUS) were used. Briefly, once MINUS and PLUS PLA probes are in close proximity, the DNA strands interact and the subsequent addition of two other circle-forming DNA oligonucleotides takes place, leading to DNA amplification reaction, detected by Texas red fluorescence. As previously reported [38], DIV10 cholinergic neurons were fixed in 4% PFA for 15 min and subjected to in situ PLA according to the manufacture’s instruction (Duolink In Situ Detection Reagents Red kit, Sigma Aldrich, DUO92008). To stain the nuclei dried coverslips were mounted with mounting medium (DUO82040). At the end of the procedure, every single event of PLA generated a fluorescent red spot. Fluorescence images were acquired on a TCS-SP5 confocal laser scanning microscope (Leica Microsystems GmbH Wetzlar, Germany) using 63 × 1.35 NA oil immersion objective. High resolution images were acquired as z-stack with a 0.5 μm z-interval (at least 10 planes), and converted to maximum projection images (LASAF software platform; Leica Microsystems). High-resolution images were analyzed by ImageJ and the number and intensity of PLA dots per neuron were measured. A manual threshold was set up and applied to all images to eliminate the background fluorescence. Objects larger than 5 μm2 were rejected, thereby effectively removing nuclei. The remaining objects were counted as PLA puncta. At least 20 neurons in five non-overlapping fields, and from 3 independent experiments, were randomly chosen by a blind observer. As a control for primary antibodies specificity, double immunolabelings with anti-TrkA and anti-pIRS_1_^Y608^ or anti-TrkA and anti-IRS_1_ antibodies (working dilution: 1:100) were run in parallel to each set of PLA experiments (Suppl. Fig. 3a and Suppl. Fig. 3b, respectively). PLA dots were undetectable following omission of the primary antibodies (Suppl. Fig. 4).

### Viability Assay

Neuronal viability was assessed by the 3-(4,5-dimethylthiazol-2-yl)-2,5-diphenyltetrazolium bromide (MTT) assay, as previously described [39].

### Statistical Analysis

Tissues from at least *n* = 3 animals per experimental group were analyzed. All experiments using primary neurons were performed at least three times independently, each in triplicate. The graphs were generated using PRISM (GraphPad Software, Inc., San Diego, CA, USA), and the data are presented as the mean ± sem. ANOVA followed by Student’s *T* test or Tukey-Kramer post hoc was used to analyze the data, depending on the number of variables and groups (Statview-SAS, Cary, NC, USA). A *p* value ≤ 0.05 was considered statistically significant.

#### Data Availability

The authors declare that all the data supporting the findings of this study are available within the article, and from the corresponding author upon reasonable request.

## Results

### Insulin Resistance Occurs in the Medial Septum of 3 Months Old 3×Tg-AD Mice

Brain insulin resistance is known to affect cognition and occurs at early stage in forebrain of different AD mouse models [19, 40]. Within the basal forebrain, the medial septum is well-known for being a primary target of AD neuropathology [4, 7, 8, 29]. However, insulin responsivity in the presymptomatic septum from AD mice has not been investigated so far.

**Fig. 1:**
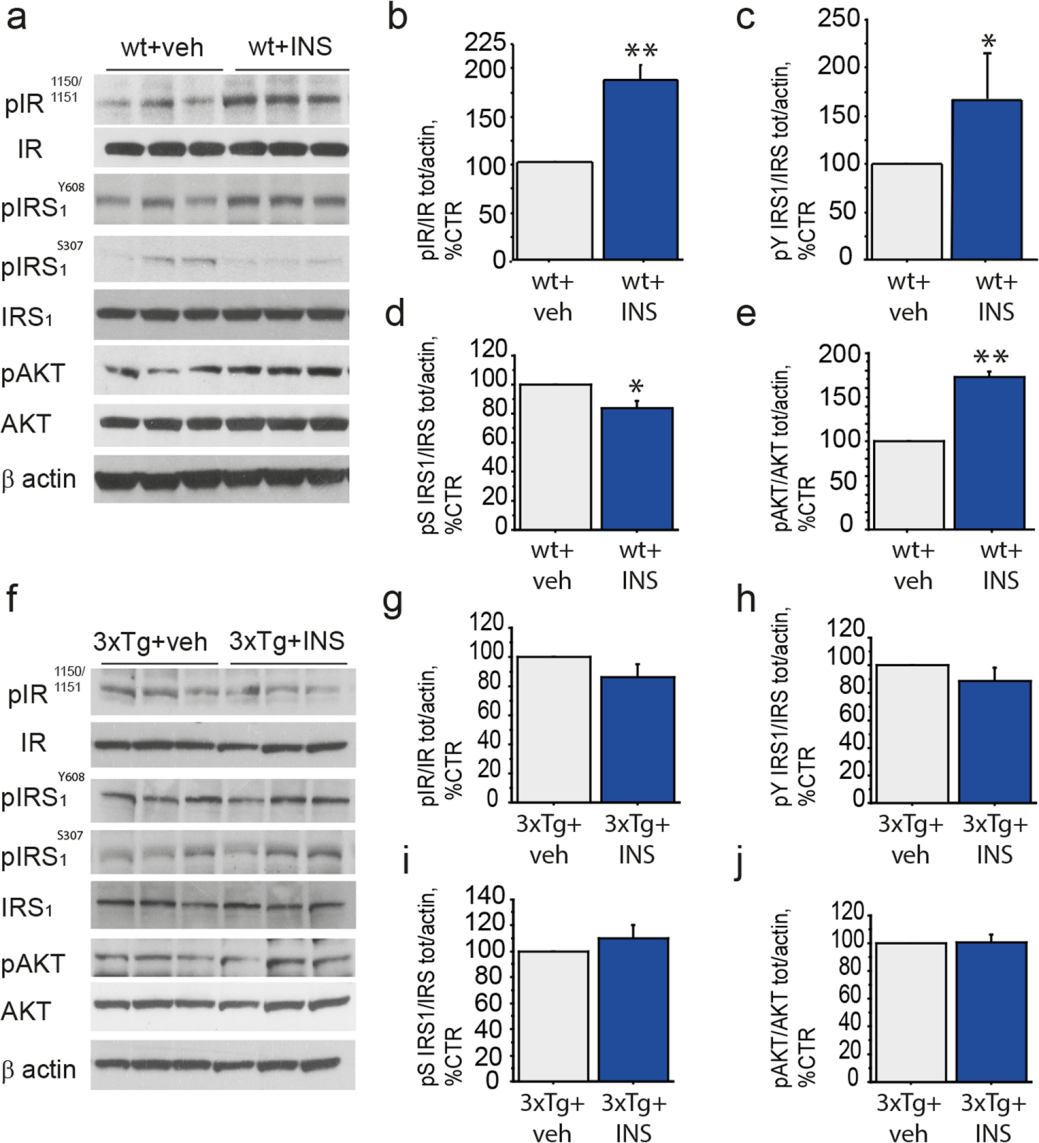
Basal forebrain system responsivity to insulin in wild-type and AD mice (**a**–**j**) Lysates of medial septum from nasal insulin and vehicle-treated 3 months old 3xTg-AD (3xTg + INS and 3xTg + veh, respectively; 30′) and age-matched wild-type (wt + INS and wt + veh, respectively; 30′) mice were analyzed by WB for the level of phosphorylated and total IR (**f**, **g** and **a**, **b**; respectively), IRS_1_^Y608^ (**f**, **h** and **a**, **c**; respectively) IRS_1_^S307^ (**f**, **i** and **a**, **d**; respectively) and AKT (**f**, **j** and **a**, **e**; respectively). The level of pIR^Y1150/1151^, pIRS_1_^Y608^ and pAKT in insulin treated mice were measured by WB using specific antibodies and β-actin, as loading control. The respective ratios over total IR, IRS_1_, and AKT levels were calculated and expressed as percentage of the vehicle treated mice. (**a**–**e**) Representative WB (**a**) and bar graphs (**b**–**e**) showing the rapid activation of the canonical insulin pathway in the medial septum of wt mice upon insulin administration, with increased phosphorylation of IR^Y1150/1151^ (182.2 ± 15.6% of wt + veh; **p < 0.01; Fig. 1b), IRS_1_^Y608^ (166.9 ± 59.4% of wt + veh; *p < 0.05; Fig. 1c) and AKT (172.6 ± 6.2% of wt + veh; **p < 0.01; Fig. 1e). (**f**–**j**) Representative WB (**f**) and bar graphs (**g**–**j**) indicate a blunted insulin response in the medial septum of 3xTg AD mice as a response to nasal stimulation. In fact, level of pIR^Y1150/1151^ (86.4 ± 8.8% of 3xTg + veh, *p* = 0.194; Fig. 1g), pIRS_1_^Y608^ (86.9 ± 9.0% of 3xTg + veh; *p* = 0.283; Fig. 1h) and pAKT (100.4 ± 6.1% of 3xTg + veh, *p* = 0.954; Fig. 1j) were unchanged following insulin treatment, a molecular state reminiscent of insulin resistance. On the other hand, insulin administration was able to downregulate the level of inhibitory pIRS_1_^S307^ in wt mice (83.9 ± 4.9% of wt + veh, *p < 0.05; Fig. 1a, d), but failed to reduce it in AD mice (109.9 ± 10.6% of 3xTg + veh, *p* = 0.402; Fig. 1f, i)

For this reason, we analyzed insulin responsivity in presymptomatic (3 months old) 3×Tg-AD and age-matched wt mice (C57/Bl6J background) by nasal administration of 0.125 IU insulin. Intranasal route of insulin delivery to the brain allows the effective bypassing of the blood-brain barrier to treat brain pathologies, AD in particular [41]. Activation of the insulin pathway was investigated by western blotting analyses of key downstream signaling molecule phosphorylation (Fig. 1a, f). In particular, the phosphorylated and total levels of IR, IRS_1_, and AKT were measured after insulin administration. Nasal insulin administration to wt mice (wt + INS) induced the rapid phosphorylation of IR^Y1150/1151^ (182.2 ± 15.6% wt + veh, **p < 0.01; Fig. 1b), IRS_1_^Y608^ (166.9 ± 59.4% wt + veh, *p < 0.05; Fig. 1c), and AKT in the medial septum (172.6 ± 6.2% wt + veh, **p < 0.01; Fig. 1e). Conversely, medial septum response to insulin administration was blunted in 3xTg-AD mice (3xTg + INS), as compared to vehicle administered 3xTg-AD mice (3xTg + veh). In particular, pIR^Y1150/1151^ (86.4 ± 8.8% of 3xTg + veh, *p* = 0.194; Fig. 1f-g), pIRS_1_^Y608^ (86.9 ± 9.0% of 3xTg + veh; *p* = 0.283; Fig. 1f, h) and pAKT (100.4 ± 6.1% of 3xTg + veh, *p* = 0.954; Fig. 1f, j) levels were unaffected by insulin. Moreover, insulin administration reduced pIRS_1_^S307^ level in wt mice (83.9 ± 4.9% of wt + veh, *p < 0.05; Fig. 1a, d) but not in AD mice (109.9 ± 10.6% of 3xTg + veh, *p* = 0.402; Fig. 1f, i). These data demonstrate that, in response to nasal insulin delivery, the basal forebrain area of the medial septum activates the canonical insulin signaling pathway in the normal mouse brain, but not in the pre-symptomatic 3×Tg-AD mice, thus showing a condition reminiscent of insulin resistance.

### Cholinergic Neurons Express IR and IRS_1_, Are Insulin Sensitive, and Respond to NGF by Activating the Insulin Pathway

Medial septum contains different type of neurons, including cholinergic, GABAergic and glutamatergic neurons [4]. Thus, to specifically investigate insulin responsivity in the cholinergic component of the medial septum, we resorted to a primary culture of cholinergic neurons.

**Fig. 2:**
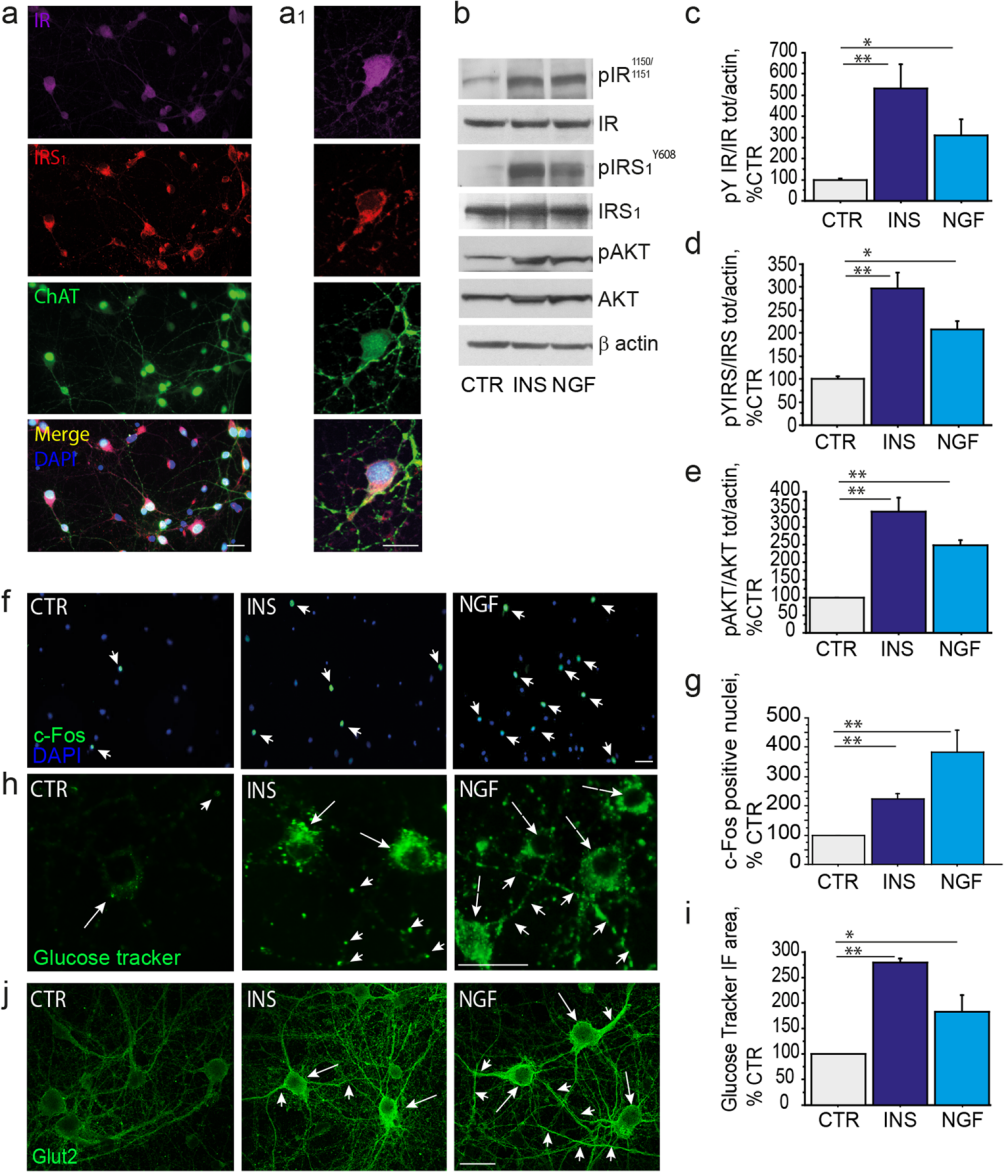
BFCN respond to insulin and NGF by inducing c-Fos expression, Glut2 translocation, and glucose uptake (**a**-**a**_1_) Triple immunolabeling (**a**, 40×) and high magnification (**a**_1_, × 100) of untreated cholinergic neurons (DIV10) using specific anti-IR (pseudocolor as *purple*), anti-IRS_1_ (*red*), and anti-ChAT (pseudocolor as *green*) antibodies clearly confirm the co-expression of IR and IRS_1_ in primary cholinergic neurons. Representative images are from at least 30 neurons. (**b**–**e**) Representative WB of pIR ^Y1150/1151^(**b**, **c**), pIRS_1_^Y608^ (**b**, **d**), and pAKT (**b**, **e**) on extracts from untreated (CTR), insulin-treated (INS, 10 nM, 30′), and NGF-treated (100 ng/ml, 30′) neurons are shown. The results are reported as a percentage of the control septal neurons. As shown, the levels of pIR^Y1150/1151^ (531.1 ± 112.4% of CTR; **p < 0.01; Fig. 2c), pIRS_1_^Y608^ (296.7 ± 35.5% of CTR; **p < 0.01; Fig. 2d), and pAKT (344.1 ± 38.8; **p < 0.01; Fig. 2e) are increased in insulin-treated cholinergic neurons, as compared to unstimulated neurons (CTR). After NGF stimulation the levels of pIR^Y1150/1151^(274.7 ± 56.5% of CTR; *p < 0.05; Fig. 2c), pIRS_1_^Y608^ (207.1 ± 18.1% of CTR; *p < 0.05; Fig. 2d) and AKT (248.9 ± 14.5% of CTR; **p < 0.01; Fig. 2e) were increased, as compared to unstimulated neurons (CTR). (**f**–**i**) The c-Fos nuclear staining and the GluTracker fluorescence analyses in untreated (CTR), insulin-treated (INS, 10 nM, 30′), and NGF-treated (NGF, 100 ng/ml, 30′) cholinergic neurons are shown. (**f**-**g**) Representative immunofluorescence images (**f**) and bar graphs (**g**) of c-Fos expression in cholinergic neurons following insulin and NGF treatments. Neurons were fixed, permeabilized and immunostained for c-Fos (*green*) and nuclei counterstained with DAPI. (**g**) The quantitative analysis of the cFos-positive nuclei/field (× 20 magnification) is reported in the graph as a percentage of the value in untreated neurons. Insulin and NGF stimulation of cholinergic neurons increased the number of c-Fos positive nuclei per field (INS, 222.7 ± 20.1% of CTR; **p < 0.01; NGF, 383.3 ± 74.1% of CTR, **p < 0.01). (**h**) Representative GluTracker immunofluorescences in cell bodies (arrows) and neurites (arrowheads) of untreated (CTR), insulin-treated (INS, 10 nM, 30′), and NGF-treated (NGF, 100 ng/ml, 30′) cholinergic neurons are shown. (**i**) The GluTracker fluorescence intensity/field was measured and reported as percentage of CTR. Insulin and NGF significantly enhanced glucose uptake in cholinergic neurons (INS, 280.3 ± 6.7% of CTR; **p < 0.01 and NGF, 183.2 ± 33.1% of CTR; *p < 0.05). (**J**) Representative confocal microscopy images of glucose transporter 2 (GLUT2; *green*) of untreated (CTR), insulin-treated (INS, 10 nM, 30′), and NGF-treated (NGF, 100 ng/ml, 30′) cholinergic neurons. As shown, both insulin and NGF treatments elevated plasma membrane localization of GLUT2 in the cell body (arrows) and axons/neurites (arrowhead). Scale Bars: a, a’, f, h, j = 25 μM

First, we studied the expression of IR and IRS_1_ in vitro*,* in rodent primary cholinergic neurons (E17, DIV10) by triple immunofluorescence labeling, using specific antibodies against the IR-β subunit, the IRS_1_ and ChAT, a marker for cholinergic neurons. We found that ChAT-positive neurons express both IR and IRS_1_ (Fig. 2a, a_1_) in neuronal culture. To assess their responsivity to insulin, cholinergic neurons (E17; DIV10) were incubated with insulin (10 nM, 30′) and the activation of IR, IRS_1_, and AKT were analyzed by WB (Fig. 2b-e). Upon insulin treatment (INS) we observed increased levels of pIR^Y1150/1151^ (531.1 ± 112.4% of CTR; **p < 0.01; Fig. 2c), pIRS_1_^Y608^ (296.7 ± 35.5% of CTR; **p < 0.01; Fig. 2d), and pAKT (344.1 ± 38.8% of CTR; **p < 0.01; Fig. 2e) compared to unstimulated neurons (CTR). Cholinergic neurons are dependent upon NGF supply for their specification and postnatal development [42, 43]. Of interest, NGF has been demonstrated to stimulate the insulin signaling in PC12-differentiated neurons [44] as well as in primary sympathetic and sensory neurons [45]. For this reason, we investigated whether NGF was able to activate the insulin pathway in cholinergic neurons. We found that the levels of pIR^Y1150/1151^ (274.7 ± 56.5% of CTR; *p < 0.05; Fig. 2c), pIRS_1_^Y608^ (207.1 ± 18.1% of CTR; *p < 0.05; Fig. 2d) and pAKT (248.9 ± 14.5% of CTR; **p < 0.01; Fig. 2e) were elevated in cholinergic neurons (DIV10) upon NGF administration (NGF; 100 ng/ml, 30′). These results indicate that cholinergic neurons are insulin responsive neurons and extend previous findings on the ability of NGF to induce the canonical activation of the insulin pathway in neuronal cells.

Then, to further characterize insulin effects on cholinergic neurons, we assayed neuronal metabolism by mean of 2d-glucose incorporation and nuclear expression of c-Fos, an Intermediate Early Gene transiently activated in response to neurotrophic stimuli in cholinergic neurons [46, 47]. Both insulin and NGF have been reported to induce c-Fos protein expression in neurons [48, 49]. As expected (Fig. 2f-g), we observed that stimulation of cholinergic neurons with both insulin and NGF increased the number of c-Fos positive nuclei per field (INS, 222.7 ± 20.1% of CTR, **p < 0.01; and NGF, 383.3 ± 74.1% of CTR; **p < 0.01, respectively). Insulin has been previously shown to promote glucose uptake in different type of CNS cells [50, 51]. We assayed glucose uptake by incubation of neurons with a bright green fluorescent 2-deoxy-glucose conjugate (GluTracker), using a cell-based commercial kit (BioVision) allowing a non-radioactive analysis of glucose metabolism by fluorescence microscopy. Both insulin and NGF significantly enhanced glucose uptake in cholinergic neurons (INS, 280.3 ± 6.7% of CTR, **p < 0.01; and NGF, 183.2 ± 33.1% of CTR, *p < 0.05; respectively; Fig. 2h-i).

Glucose is taken up by glucose transporters (Glut) following stimulus-dependent translocation of Glut from the cytosol to the plasma membrane. In particular, Glut2 is a glucose sensor and is widely expressed in the brain, including the medial septum [52]. Glut4 is the insulin-responsive Glut isoform in the peripheral organs and is present in several brain tissues [53]. However, their role in glucose uptake in cholinergic neurons has not been established. In order to assess whether Glut2 and/or Glut4 are implicated in glucose uptake in cholinergic neurons, we immunolabeled them with specific anti-Glut2 and anti-Glut4 antibodies. Glut2 staining was found to be dispersed in the cytosol in starved neurons (CTR), while treatment with NGF induced a strong Glut2 re-localization at the plasma membrane (Fig. 2j) at both cell bodies (arrows) and neurites (arrowheads). By contrast, neither insulin nor NGF were able to induce translocation of Glut-4 to the plasma membrane, despite this glucose transporter is expressed in primary cholinergic neurons (Suppl. Fig. 1e).

### Insulin Resistance Can Be Induced in Primary Cholinergic Neurons by Chronic High Insulin and It Is Ameliorated by NGF

**Fig. 3:**
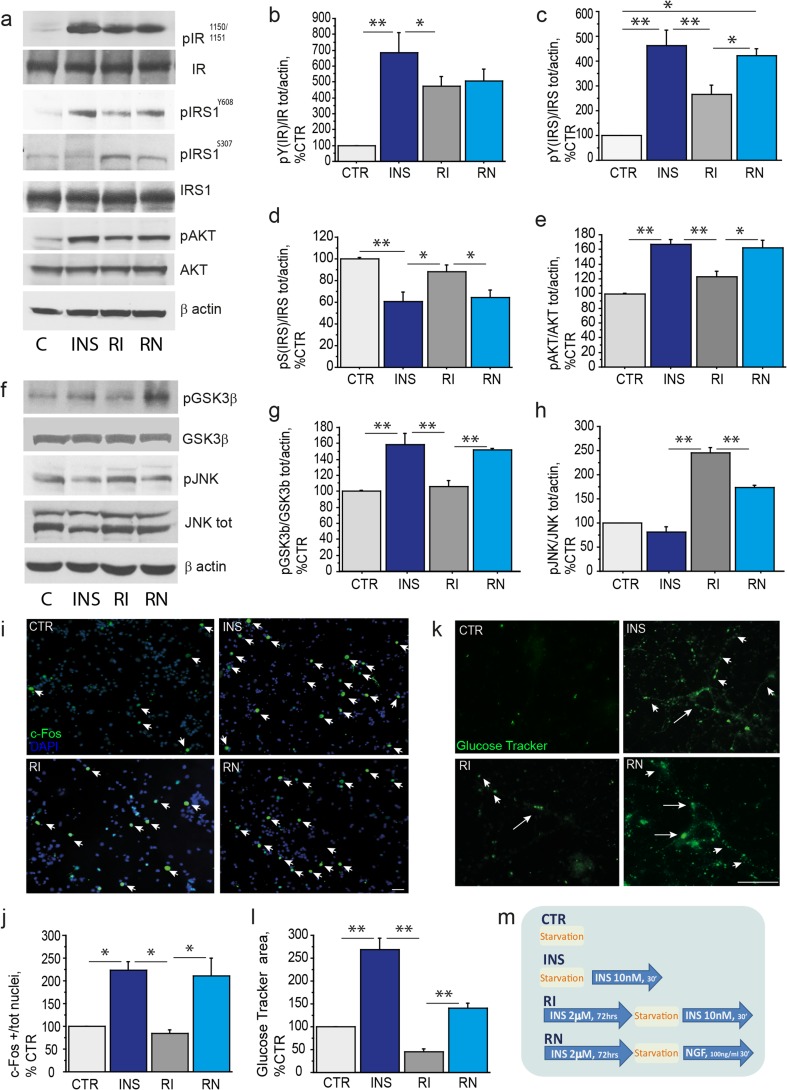
Chronic high insulin induces insulin resistance in cholinergic neurons and can be improved by NGF. (**a**–**l**) Insulin resistance experimental data and **(m)** the related experimental paradigm scheme. (**a**–**e**) A representative WB (**a**) and relative bar graphs (**b**-**e**) of extracts from untreated (CTR), insulin-treated (INS), insulin-resistant (RI), and insulin-resistant and NGF-treated (RN) cholinergic neurons, using antibodies against pIR^Y1150/1151^, IR, pIRS_1_^Y608^, pIRS_1_^S307^, IRS_1_, pAKT, AKT with β-actin as loading control, are shown. The insulin treatment increases tyrosine phosphorylation of IR (683.8±127.1% of CTR; INS vs CTR,**p <  0.01; Fig. 3a-b), IRS_1_^Y608^ (462.2 ± 63.6% of CTR; INS vs CTR,**p < 0.01; Fig. 3a, c) and AKT (166.9±6.1% of CTR; INS vs CTR,**p < 0.01; Fig. 3a, e) in control cholinergic neurons, as expected. Of note, insulin downregulates the inhibitory IRS_1_^S307^ (60.3±9.1% of CTR; INS vs CTR,**p < 0.01; Fig. 3a, d) and inactivates GSK3β by S9 phosphorylation (158.6±13.5% of CTR; INS vs CTR,**p < 0.01; Fig.3f-g), while it has no effect on JNK activation (81.9±10.9% of CTR; INS vs CTR, p=0.24; Fig. 3f, h). After chronic high insulin, the level of tyrosine phosphorylation of the IR (RI, 472.6 ± 63.5% of CTR; RI vs INS, *p < 0.05; Fig. 3a, b) and the IRS_1_^Y608^ (RI, 264.9 ± 37.8% of CTR; RI vs INS, **p < 0.01; Fig. 3a, c), as well as activation of AKT (RI, 122.9 ± 7.7% of CTR; RI vs INS, **p < 0.01; Fig. 3a, e) are reduced in cholinergic neurons, while the serine phosphorylation of IRS_1_ was significantly elevated, as compared to INS (RI, 87.9 ± 6.3% of CTR; RI vs INS, *p < 0.05; Fig. 3a, d). The ratio was calculated over the total protein levels and expressed as percentage of the control. NGF treatment restored tyrosine phosphorylation of IRS_1_ (RN, 421.8 ± 27.6% of CTR, RN vs RI, *p < 0.05; Fig. 3a, c) and of AKT (RN, 161.8 ± 11.1% of CTR; RN vs RI, *p < 0.05; Fig. 3a, e), and reduced serine phosphorylation of IRS_1_ (RN, 64.5 ± 7% of CTR; RN vs RI, *p < 0.05; Fig. 3a, d). Any effect was found on the pIR levels (RN, 505.49 ± 76.9% of CTR; RN vs RI, *p* = 0.76; Fig. 3a-b). (**f-h**) Lysates of primary septal neurons from unstimulated (CTR), insulin-treated (INS), insulin-resistant (RI), and insulin-resistant NGF-treated (RN) were analyzed by WB, using specific antibodies against pGSK3β, pJNK, total GSK3β, and total JNK with β-actin as loading control. The data are reported as percentage of control in the graph (Fig. 3g-h). The levels of pGSK3β are reduced (RI, 105.9 ± 7.9% of CTR; RI vs INS, **p < 0.01; Fig. 3f-g) and pJNK is augmented (RI, 244.5 ± 12.1% of CTR; RI vs INS, **p < 0.01; Fig. 3f, h) in insulin resistant cholinergic neurons. In turn, NGF treatment restored GSK3β (RN, 151.6 ± 1.9% of CTR; RN vs RI, **p < 0.01; Fig. 3f-g) and JNK (RN, 173.7 ± 3.9% of CTR; RN vs RI, **p < 0.01; Fig. 3f, h) phosphorylation as compared to RI. (**i-l**) The c-Fos nuclear staining (**i-j**) and GluTracker labeling (**k-l**) of CTR, INS, RI, and RN cholinergic neurons are shown. (**i**) Cholinergic neurons were fixed, permeabilized and immunostained for c-Fos (*green*) and nuclei stained by DAPI (*blue*). (**j**) The quantitative analysis of the c-Fos positive nuclei/field (× 20 magnification) is reported in the graph as a percentage of the value in control neurons. (**k**) After the appropriate treatment (m), the cholinergic neurons were incubated with the GluTracker mix, fixed and mounted on glass slides. (**l**) The quantitative analysis of GluTracker fluorescence intensity was calculated and reported as percentage of control. Chronic high insulin significantly reduces insulin-driven c-Fos nuclear staining (84.6 ± 7.4% of CTR; RI vs INS: *p < 0.05; Fig. 3i-j) and neuronal glucose uptake (44.9 ± 7.1% of CTR; RI vs INS, **p < 0.01; Fig. 3k-l), as compared to control conditions. Noteworthy, NGF stimulation rescued the number of c-Fos positive nuclei (210.2 ± 40.3% of CTR; RN vs RI: *p < 0.05; Fig. 3i-j), as well as level of glucose uptake (140.2 ± 12% of CTR; RN vs RI: **p < 0.01; Fig. 3k-l) in insulin resistant cholinergic neurons. (m) CTR: DIV 13 starved and untreated neurons; INS: starved and insulin-treated (10 nM, 30′) DIV13 neurons; RI: DIV10 neurons exposed to chronic high insulin (2 μM, 72 h), starved at DIV13 and then exposed to 10 nM insulin (30′); RN = DIV10 neurons exposed to chronic high insulin (2 μM, 72 h), starved at DIV13 and then exposed to NGF (NGF 100 ng/ml, 30′). Starvation consisted in replacement of neurobasal medium without B27 (90′) to avoid confounding effect from B27 derived insulin. Scale bars: i,k = 25 μM

Brain insulin resistance is defined as a state of reduced brain responsivity to insulin stimulation. It is characterized by decreased tyrosine phosphorylation of IR and IRS_1_ and the concomitant increase in serine phosphorylation of IRS_1_, especially at S307 [20]. Chronic high insulin has been already reported to induce a state mimicking insulin resistance in cortical neurons in vitro [54]. Thus, in order to study insulin resistance in primary cholinergic neurons, the latter were treated with 2μM insulin for 72 h (from DIV10 to DIV13). Acute stimulation with 10 nM insulin (30′) was performed to assess insulin responsivity following chronic high insulin exposure. The experimental scheme of the chronic high insulin protocol is reported in Fig. 3m.

The levels of pIR^Y1150/1151^, pIRS_1_^Y608^, pIRS_1_^S307^, and pAKT were measured and reported as percentage of control (CTR) in insulin treated (INS; 10 nM, 30′) and insulin resistant (RI; 2 μM INS, 72 h followed by 10 nM INS, 30′) cholinergic neurons. Insulin promotes phosphorylation of IR (683.8±127.1% of CTR; INS vs CTR,**p < 0.01; Fig. 3a-b), IRS_1_ (462.2 ± 63.6% of CTR; INS vs CTR,**p < 0.01; Fig. 3a, c) and AKT (166.9±6.1% of CTR; INS vs CTR,**p < 0.01; Fig. 3a, e), as expected. Of note, insulin downregulates two inhibitory molecules of the insulin pathway, IRS_1_^S307^ (60.3±9.1% of CTR; INS vs CTR,**p < 0.01; Fig. 3a, d) and GSK3β (158.6±13.5% of CTR; INS vs CTR,**p < 0.01; Fig. 3f-g). No effect is observed on phosphorylation of JNK in control cholinergic neurons (81.9±10.9% of CTR; INS vs CTR, p=0.24; Fig. 3f, h). As shown in Fig. 3a-e, IR (472.6 ± 63.5% of CTR; RI vs INS, *p < 0.05; Fig. 3a, b), IRS_1_^Y608^ (264.9 ± 37.8% of CTR; RI vs INS, **p < 0.01; Fig. 3a, c), and AKT (122.9 ± 7.7% of CTR; RI vs INS, **p < 0.01; Fig. 3a, e) failed to be activated, while the inhibitory serine phosphorylation of IRS_1_ was significantly elevated (RI, 87.9 ± 6.3% of CTR; RI vs INS, *p < 0.05; Fig. 3a, d) following chronic high insulin. Brain insulin resistance is characterized by increased activation of GSK3β and JNK, two critical kinases implicated in IRS_1_ inhibition [22]. To further characterize our experimental model, we analyzed the levels of GSK3β and JNK in cholinergic neurons (Fig. 3f). We found that phosphorylation of GSK3β at serine 9 was reduced in cholinergic neurons following chronic high insulin (RI, 105.9 ± 7.9% of CTR; RI vs INS, **p < 0.01; Fig. 3f-g), while JNK phosphorylation was dramatically elevated in the same conditions (RI, 244.5 ± 12.1% of CTR; RI vs INS, **p < 0.01; Fig. 3f, h). Overall, the in vitro paradigm developed effectively mimics insulin resistance at the molecular level in cultured cholinergic neurons.

Further, we asked whether NGF is able to counteract these molecular changes. For this purpose, we treated insulin resistant cholinergic neurons (DIV13) with NGF. As shown in Fig. 3 (a-e), NGF restores tyrosine phosphorylation of IRS_1_ (RN, 421.8 ± 27.6% of CTR; RN vs RI, *p < 0.05; Fig. 3a, c) and reduces serine phosphorylation of IRS_1_ (RN, 64.5 ± 7% of CTR; RN vs RI, *p < 0.05; Fig. 3a, d). NGF also stimulated AKT (RN, 161.8 ± 11.1% of CTR; RN vs RI, *p < 0.05; Fig. 3a, e), recovered the inhibitory phosphorylation of GSK3β (RN, 151.6 ± 1.9% of CTR; RN vs RI, **p < 0.01; Fig. 3f, g), and downregulated JNK (RN, 173.7 ± 3.9% of CTR; RN vs RI **p < 0.01; Fig. 3f, h). No statistically significant difference was observed as for the activation of IR upon NGF treatment of insulin resistant neurons (RN, 505.49 ± 76.9% of CTR; RN vs RI, *p* = 0.76; Fig. 3a, b).

Significantly, chronic high insulin also affected nuclear c-Fos expression (RI, 84.6 ± 7.4% of CTR; RI vs INS, *p < 0.05; Fig. 3i-j), and reduced glucose uptake (RI, 44.9 ± 7.1% of CTR; RI vs INS, **p < 0.01; Fig. 3). Noteworthy, NGF stimulation restored the number of c-Fos positive nuclei (RN, 210.2 ± 40.3% of CTR; RN vs RI, *p < 0.05; Fig. 3i-j), as well as glucose uptake (RN, 140.2 ± 12% of CTR; RN vs RI, **p < 0.01; Fig. 3k-l) in insulin resistant cholinergic neurons. Thus, our results confirm and extend previous reports on the neuroprotective effect of NGF in the medial septum [5, 55] pinpointing the ability of the NGF pathway to stimulate the insulin signaling and glucose homeostasis in physiological conditions and also to ameliorate insulin resistance in cultured cholinergic neurons.

### Stimulation of the Insulin Pathway and Neuronal Metabolism by NGF Is Achieved Through IRS_1_ Activation and Binding to TrkA

**Fig. 4:**
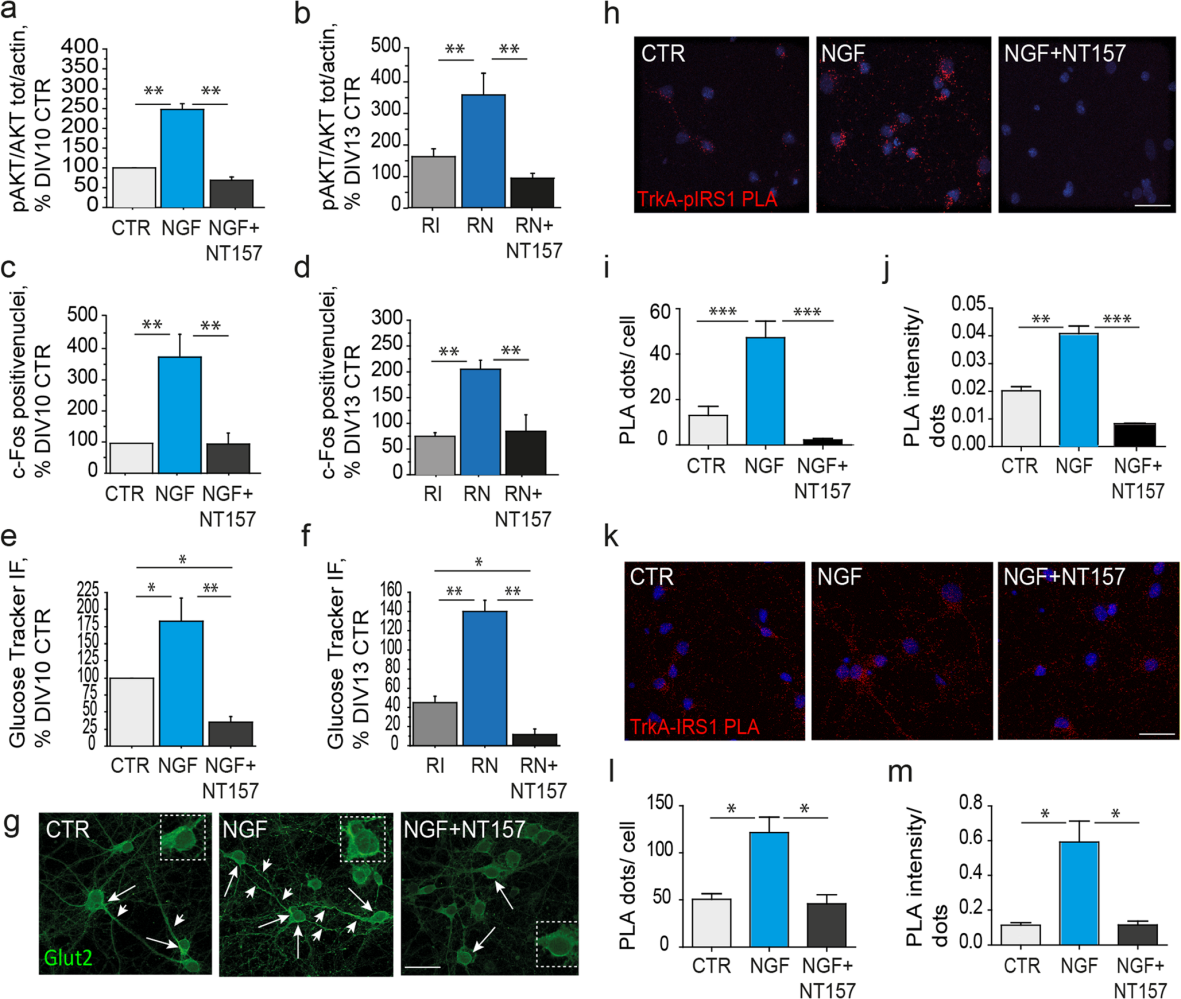
Rescue of Insulin resistance upon NGF stimulation occurs mainly by IRS_1_ activation and TrkA/IRS_1_ binding. (**a**–**f**) Densitometric analyses of AKT (**a**, **b**; respectively), c-Fos staining (**c**, **d**; respectively) and Glucose uptake (**e**, **f**; respectively) in control (**a, c, e)** and insulin resistant (**b, d, f**) cholinergic neurons. (**a-b**) Levels of pAKT, total AKT, and β-actin are reported (**a**) in control (CTR), NGF-treated (NGF), and NGF-treated neurons preincubated with the specific IRS inhibitor NT157 (NGF + NT157), and (**b**) in RI, RN, and RN following incubation with NT157 (RN + NT157). NGF increases AKT activation in control (NGF, 248.9 ± 14.5% of CTR; **p < 0.01; Fig. 4a) and insulin resistant (RN, 359.4 ± 68.9% of CTR; **p < 0.01; Fig. 4b) cholinergic neurons, but fails to modulate it in presence of NT157 in both control (NGF + NT157, 68.6 ± 9.3% of CTR; **p < 0.01; Fig. 4a) and insulin resistance conditions (RN + NT157, 93.1 ± 15.1% of CTR; **p < 0.01, Fig. 4b). (**c-d**) Quantitative analysis of the c-Fos positive nuclei/field (× 20 magnification) in (**a**) CTR, NGF, NGF-treated neurons in presence of a specific IRS inhibitor NT157 (NGF + NT157), and in (**d**) insulin-resistance (RI), insulin-resistance NGF-treated (RN), and insulin-resistance NGF-treated in presence of a specific IRS inhibitor NT157 (RN + NT157). NGF elevates the number of c-Fos expressing neurons in control (383.3 ± 74.1% of CTR; **p < 0.01; Fig. 4c), as well as in insulin resistant cholinergic neurons (210 ± 40.2% of CTR; **p < 0.01; Fig. 4d), but its effect is lost following IRS inhibition by NT157 both in control (96.1 ± 35.5% of CTR; NGF + NT157 vs NGF, **p < 0.01; Fig. 4c) and insulin resistant conditions (96 ± 35.7% of CTR; RN + NT157 vs RN, **p < 0.01; Fig. 4d). (**e-f**) Quantitative analysis of glucose uptake in (**e**) control untreated (CTR), NGF-treated (NGF), NGF-treated with NT157 (NGF + NT157), and in (**f**) insulin-resistant (RI), insulin-resistant NGF-treated (RN), and insulin-resistant NGF-treated neurons preincubated with NT157 (RN + NT157). NGF stimulated glucose uptake in control (183.2 ± 33% of CTR, *p < 0.05; Fig. 4e) and insulin resistant neurons (140.2 ± 12% of CTR, **p < 0.01; Fig. 4f), but lost its efficacy after preincubation with NT157 in control (35.3 ± 7.5% of CTR; NGF + NT157 vs NGF, **p < 0.01; Fig. 4e) and insulin resistant (11.3 ± 6.5% of CTR; RN + NT157 vs RN, **p < 0.01; Fig. 4f) conditions, where glucose uptake is lower than in RI condition (RN + NT157 vs RI, *p < 0.05). The fluorescence intensity was calculated as percentage of control (CTR) and reported as mean ± sem. CTR neurons were starved DIV10 cholinergic neurons in control conditions and starved DIV13 neurons in insulin resistant experiments. **(g)** Glut2 immunofluorescent labeling in cholinergic neurons (DIV10) treated with NGF (100 ng/ml, 30′) with and without preincubation with NT157 (10 μM, 2 h). Specific anti-Glut2 antibodies were used and detected Glut2 immunoreactivity mainly in the cytosolic compartment of both the cell bodies (arrows) and neurites (arrowheads) in untreated neurons (CTR). NGF incubation (NGF) drives plasma membrane translocation of Glut2, both at the cell bodies (arrows) and neurites (arrowheads) in absence of NT157. While NT157 preincubation (NGF + NT157) hampers the effect of NGF on Glut2 translocation, Glut2 labeling being mainly cytosolic, as observed in CTR neurons. **(h,k)** Detection of TrkA-pIRS_1_^Y608^ (h) and TrkA-IRS_1_ (k) interactions by PLA assay in cholinergic neurons (DIV10) treated with NGF (100ng/ml, 30'), with and without preincubation with NT157 (10μM, 2 h). Neurons were fixed with PFA, blocked with normal donkey serum (10%, 1 h, RT) and incubated (ON, 4 °C) with a combination of mouse anti-Trk and rabbit anti-pIRS_1_^Y608^ or mouse anti-Trk and rabbit anti-total IRS_1_. Detection and amplification of single events of interaction by a single dot (*red*) was achieved by the PLA assay (PLA Duoset, Sigma). Nuclei were counterstained with DAPI. PLA staining was analyzed by an observer blind of the experimental group and representative picture were randomly chosen. **(i-j)** Quantification of the number (i) and intensity (j) of the TrkA-pIRS_1_^Y608^ PLA signal dots in primary neurons untreated (CTR, 13 ± 4 dots/neuron and 0.02 ± 0.002 intensity/dot), treated with NGF (NGF, 47.2 ± 7.3 dots/neuron, ***p < 0.001 and 0.04 ± 0.002 intensity/dot, **p < 0.01) or NGF following NT157 preincubation (NGF + NT157, 2.2 ± 0.65 dots/neuron; NGF + NT157 vs NGF, ***p < 0.001; and 0.008 ± 0.0002 intensity/dot, NGF + NT157 vs NGF, ***p < 0.001). **(k-l)** Quantification of the number (**k**) and intensity (**l**) of the TrkA-IRS_1_ PLA signal dots in primary neurons untreated (CTR, 50.6 ± 5.9 dots/neuron and 0.11 ± 0.015, intensity per dot), NGF treated (121.5 ± 16.4 dots/neuron, *p < 0.05; 0.59 ± 0.12 intensity/dot, *p < 0.05), or NGF treated following NT157 preincubation (NGF + NT157, 45.8 ± 9.6 dots/neuron NGF + NT157 vs NGF; *p < 0.05; 0.12 ± 0.02 intensity per dot, NGF + NT157 vs NGF *p < 0.05). The quantitative analysis of the PLA fluorescent signal was performed in five different fields, for a total of 20 to 30 neurons and was expressed as mean ± sem. Integrated intensity per dot is expressed in arbitrary unit (AU) and reported as mean ± sem. Scale bars: g = 50 μm; h,k = 25 μm. CTR neurons in **a, c, e** are DIV10 untreated neurons; CTR neurons in **b, d, f** are DIV13 untreated neurons

We demonstrated the ability of NGF to improve insulin resistance, mainly stimulating IRS_1_ in primary cholinergic neurons. This is in line with the findings that several members of the receptor tyrosine kinases (RTK) superfamily like IGFI/II receptors, the BDNF receptor TrkB, and ErbB can trans-activate the insulin pathway by enhancing IRS_1_ and the downstream insulin pathway (e.g., AKT, GSK3β) [56]. Accordingly, we asked whether the rescue effect of NGF on survival and metabolism in insulin resistant cholinergic neurons depends on IRS_1_. To address this question, we incubated cholinergic neurons with NGF with and without previous treatment with the selective IRS inhibitor NT157. First, NT157 (10 μM, 2 h) was found to effectively inhibits IRS_1_ (Suppl. Fig. 2a) and AKT (Suppl. Fig. 2b) phosphorylation under insulin stimulation. Then, we analyzed AKT activation, c-Fos expression and glucose uptake in control (Fig. 4a, c, e) and insulin resistant (Fig. 4b, d, f) cholinergic neurons. We found that IRS inhibition by NT157 abolished the NGF effect on pAKT levels in control (NGF + NT157, 68.6 ± 9.3% of DIV10 CTR; NGF + NT157 vs NGF, **p < 0.01, Fig. 4a and Suppl. Fig. 2g) and in insulin resistant (RN + NT157, 93.1 ± 15.1% of DIV13 CTR; RN + NT157 vs RN; **p < 0.01, Fig. 4b and Suppl. Fig. 2h) neurons. A similar trend was observed for c-Fos expression, indicating that NGF effect was lost both in control (NGF + NT157, 96.1 ± 35.5% of DIV10 CTR; NGF + NT157 vs NGF, **p < 0.01; Fig. 4c) and insulin resistant conditions (RN + NT157, 96.0 ± 35.7% of DIV13 CTR; RN + NT157 vs RN, **p < 0.01; Fig. 4d). Further, following incubation with the specific IRS inhibitor NT157, NGF was unable to rescue glucose uptake both in control (NGF + NT157, 35.3 ± 7.5% of DIV10 CTR; NGF + NT157 vs NGF, **p < 0.01; Fig. 4e) and insulin resistant (RN + NT157, 11.3 ± 6.5% of DIV13 CTR; RN + NT157 vs RN, **p < 0.01; Fig. 4f) cholinergic neurons. Of note, NT157 treatment not only abolished NGF effect on glucose uptake in insulin resistance, but it also further reduced glucose metabolism, as compared to control (NGF + NT157 vs DIV10 CTR, **p < 0.01; Fig. 4e) and insulin resistant conditions (RN + NT157 vs RI; *p < 0.05; Fig. 4f), suggesting that most of the glucose uptake likely occurs under the IRS control in cholinergic neurons. Of note, NT157 incubation did not affect neuronal viability, as assessed by the MTT test (*p* = 0.16; Suppl. Fig. 2c). To further characterize the role of IRS_1_ in NGF-mediated control of glucose metabolism, we immunolabeled DIV10 cholinergic neurons with NGF in the presence of NT157 with antibodies against Glut2 and Glut4. We found that NT157 abolished the observed NGF effect on Glut2 translocation to the plasma membrane (Figs. 2j and 4g), while no effect was observed for Glut4 (Suppl. Fig. 2i). These data demonstrate that the NGF effect on neuronal metabolism in control and insulin resistant cholinergic neurons is abolished by the IRS inhibitor NT157, and it thus requires IRS activation.

NGF stimulation of insulin pathway via IRS_1_ transactivation has been previously reported in cancer cells, and occurs upon IRS_1_ recruitment by the NGF receptor TrkA [19, 28]. For this reason, we asked whether TrkA is able to bind and recruit IRS_1_ also in our experimental conditions. To investigate the existence of a TrkA/IRS_1_ complex in cholinergic neurons we resorted to the Proximity Ligation Assay (PLA) approach. PLA is a well-described technique providing enough sensitivity to evaluate endogenous protein’s close proximity in native conditions. PLA allows visualization of single events of protein interaction by generating single immunofluorescent dots.

Thus, by using proper antibody combinations, TrkA/pIRS_1_ (Fig. 4h) and TrkA/IRS_1_^Y608^ (Fig. 4k) complexes were visualized and PLA dot number and intensity per neuron were measured in untreated and NGF-treated neurons (100ng/ml, 30') with or without NT157 (10μM, 2 h) preincubation. A faint TrkA/pIRS_1_^Y608^ PLA signal was observed throughout the entire neuron, from the cell body to the distal parts of the neurites in few control neurons (CTR; Fig. 4h). Treatment with NGF stimulates the interaction between TrkA and the active form of IRS_1_. Interestingly, PLA signal was negligible in NGF-NT157 treated neurons (Fig. 4h). The number of dot/neuron augmented upon NGF treatment (NGF: 47.2 ± 7.3 vs CTR: 13 ± 4 dots/neuron; ***p < 0.001, Fig. 4i), while NGF + NT157 treated neurons displayed a very low PLA signal (2.20 ± 0.65 dots/neuron; ***p < 0.001, Fig. 4i). Moreover, a strong increase in the TrkA/pIRS_1_^Y608^ PLA intensity was observed under NGF stimulation (NGF: 0.040 ± 0.002 vs CTR: 0.023 ± 0.002 dot intensity/neuron; **p < 0.01, Fig. 4j). Cholinergic neurons treated with NGF + NT157 displayed very low PLA intensity signal (0.008 ± 0.000 dot intensity/neuron; ***p < 0.001, Fig. 4j). As opposite to TrkA/pIRS_1_^Y608^ PLA signal, TrkA/IRS_1_ PLA was detectable in control neurons (CTR), and strongly increased after NGF treatment, while NT157 preincubation significantly reduced the NGF-driven effect (Fig. 4k). In fact, the number of TrkA/IRS_1_ PLA dots were significantly augmented by NGF (121.50 ± 0.60 vs 50.60 ± 0.10 CTR dots/neuron, *p < 0.05; Fig. 4l). PLA intensity per dot was also augmented after NGF treatment (0.600 ± 0.100 vs 0.100 ± 0.020 CTR, arbitrary units,*p < 0.05, Fig. 4m). Importantly, TrkA/IRS_1_ PLA dots number and intensity were affected (45.80 ± 9.60 and 0.100 ± 0.020; Fig. 4l and 4m, respectively) by NT157 pretreatment (NGF + NT157), confirming the requirement of IRS activation for the TrkA-IRS_1_ interaction to occur. Taken together, data from our PLA assay experiments strongly support the existence of the TrkA/IRS_1_ complex modulated by NGF in cholinergic neurons and dependent upon tyrosine phosphorylation of IRS_1_. Notably, neither total IRS1 (*p* = 0.9) nor TrkA (*p* = 0.56) levels were affected by NT157 in our experimental conditions (Suppl. Fig. 2d-f).

### NGF Nasal Delivery Rescues the Insulin Pathway and Increases ChAT in the Septum of 3×Tg-AD Mice

**Fig. 5:**
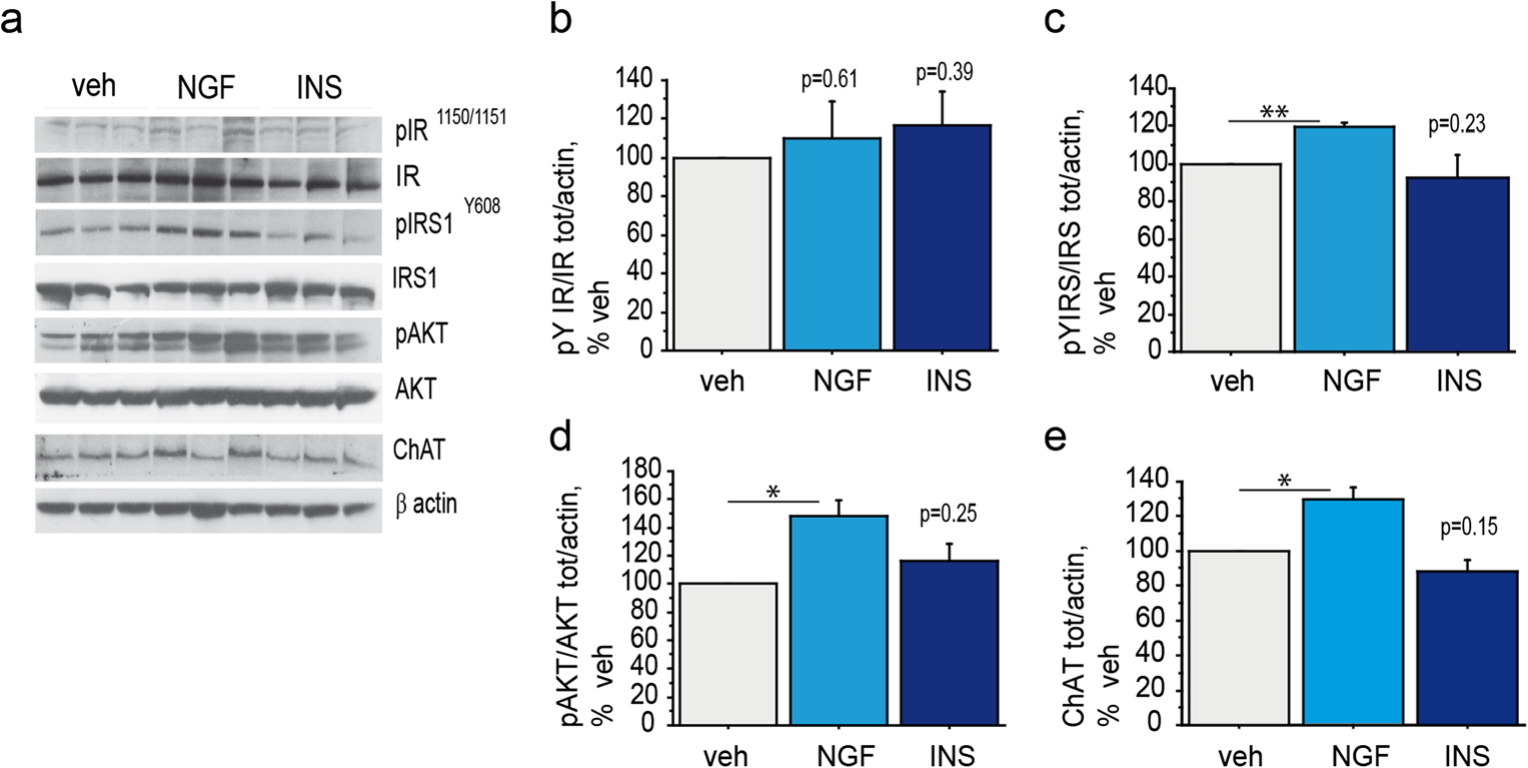
Rescue of Insulin resistance in the medial septum upon NGF nasal administration to 3xTg-AD mice (**a**–**e**) Representative western blots (WB) of pIR ^Y1150/1151^ (**a**–**b**), pIRS_1_^Y 608^ (**a**, **c**), pAKT (**a**, **d**) and ChAT (**a**, **e**) on septal extracts from 3xTg-AD mice (*n* = 4–6 mice/experimental group) nasally administered with NGF (NGF, 40 μg/mouse), insulin (INS, 0.125 IU/mouse) or vehicle (veh). Nasal delivery of NGF resulted in increased phosphorylation levels of IRS^Y608^ (119.7 ± 1.9% of veh, **p < 0.01; Fig. 5a, c), AKT (148.4 ± 11.3% of veh, *p < 0.05; Fig. 5a, d), and ChAT (125.8 ± 5.8% of veh, *p < 0.05; Fig. 5a, e) in the medial septum of AD mice. No statistically significant effect of NGF treatment was observed on IR activation (110.5 ± 18.6% of veh, *p* = 0.61; Fig. 5a-b). Insulin treatment did not result in phosphorylation of IR^Y1150/1151^ (116.6 ± 17.3% of veh, *p* = 0.39; Fig. 5a-b), IRS_1_^Y608^ (89.8 ± 12.4% of veh, *p* = 0.23; Fig. 5a, c), and AKT (116.6 ± 12.2% of veh, *p* = 0.25; Fig. 5a, d). Further, insulin showed any effect on ChAT expression (88.4 ± 6.5% of veh, *p* = 0.15; Fig. 5a, e)

To assess whether insulin resistance of cholinergic neurons can be rescued from NGF also in vivo, young adult 3×Tg-AD mice were nasally administered with NGF (NGF, 40 μg/mouse), insulin (INS, 0.125 IU/mouse) or vehicle (veh) by bilateral nasal drop (2.5 μl/nostril) and sacrificed 30 min later. Activation of the early (IR and IRS_1_) and late (AKT) signaling molecules of the insulin pathway, as well as the expression of the cholinergic marker, ChAT, were investigated (Fig. 5a-e). Nasal delivery of NGF resulted in increased IRS_1_^Y608^ (119.7 ± 1.9% of veh, **p < 0.01; Fig. 5a, c) and AKT (148.4 ± 11.3% of veh, *p < 0.05; Fig. 5a, d) phosphorylation. The expression of ChAT was also augmented by NGF (125.8 ± 5.8% of veh, *p < 0.05; Fig. 5a, e), while no effect was observed on IR activation (110.5 ± 18.6% of veh, *p* = 0.61; Fig. 5a-b). Insulin administration did not result in the activation of the insulin pathway, as observed by phosphorylation of IR^Y1150/1151^ (116.6 ± 17.3% of veh, *p* = 0.39; Fig. 5a-b), IRS_1_^Y608^ (89.8 ± 12.4% of veh, *p* = 0.23; Fig. 5a, c) and AKT (116.6 ± 12.2% of veh, *p* = 0.25; Fig. 5a, d), confirming previously shown results (Fig. 1). Also, ChAT expression was unaffected by insulin (88.4 ± 6.5% of veh, *p* = 0.15; Fig. 5a, e).

## Discussion

To the best of our knowledge, this is the first study addressing medial septum sensitivity to insulin stimulation in wt and AD mice. Here we present in vivo evidence of medial septum sensitivity to insulin upon nasal administration, with the activation of the canonical insulin pathway in wild-type mice (Fig. 1a-e).

Although the brain has been long time considered an insulin independent organ, compelling evidences show that insulin not only is locally produced, but it also induces glucose uptake, has neuroregulatory properties on nutrient intake in the brain, modulating learning and memory, and controlling body energy intake [12, 41, 57]. Accordingly, IR and IRS mRNA and protein are co-expressed in several brain tissues, like the hippocampus, the cortex, the hypothalamus, including the basal forebrain [13, 58, 59].

Conversely, insulin treatment was unable to elicit the activation of the key insulin signaling molecules (IR-IRS_1_-AKT) in the basal forebrain of 3×Tg-AD mice (Fig. 1f-j). Nasal administration is a well characterized and extensively used method of brain delivery [60], and nasal insulin stimulation has been shown to be the paradigm of choice to highlight brain insulin resistance [20]. Thus, insulin failure to activate the insulin pathway in the medial septum of 3×Tg-AD mice suggests that these mice developed a condition reminiscent of brain insulin resistance. Moreover, insulin resistance has been observed at 3 months of age, while synaptic deficits and cognitive impairments are not detectable before 5–6 months of age in 3×Tg-AD mice [32, 61, 62]. Neocortical insulin resistance and systemic deregulation of glucose metabolism can be detected only 2–3 months later, before the appearance of blood hyperglicemia and peripheral insulin resistance [31, 62], pinpointing that insulin resistance in the medial septum develops during the AD pre-symptomatic phase of 3×Tg-AD mice [63, 64].

The perturbation of the insulin pathway has been shown to affect cholinergic phenotype and synaptic functions in cultured neurons [24]. Thus, the effects of insulin and insulin resistance on the BFCS and related cognition is of great relevance for ameliorating insulin resistance-dependent impairment of cognition observed in both neurodegenerative disorders (e.g. AD) and metabolic diseases (e.g. Type 2 Diabetes, T2D).

In order to investigate a crosstalk between the insulin signaling and neuronal metabolism in BFCN, we cultured neurons from the rodent septum following a well established and characterized in vitro model culture [35, 36]. We observed that cultured cholinergic neurons express both IR and IRS (Fig. 2a), as already observed in vivo in rodents [14]. Moreover, they activate the canonical insulin pathway in response to insulin stimulation by increasing levels of pIR^Y1151/1150^ (Fig. 2b-c; 500% of CTR), pIRS_1_^Y608^ (Fig. 2b, d; 300% of CTR), and pAKT (Fig. 2b, e; 350% of CTR). In line with this, glucose uptake was significantly augmented (Fig. 2h-i; 275% of CTR) and c-Fos expression increased in response to insulin stimulation (Fig. 2f-g; 200% of CTR). These findings show that BFCN are insulin sensitive and their neuronal metabolism and activity are under insulin control.

Next, we set up a novel chronic high insulin-based cellular paradigm to specifically address insulin resistance in cholinergic neurons and found that the IR-IRS-AKT signaling pathway is significantly downregulated in cholinergic neurons (Fig. 3). In turn, the activity of the main IRS_1_ serine kinases, GSK3β and JNK1/2, are upregulated (Fig. 3f-h), while c-Fos expression and glucose uptake are significantly reduced (Fig. 3i-l). These findings indicate that this in vitro paradigm does recapitulate the main features of insulin resistance at the cellular level, and may represent a valuable tool for the screening of insulin sensitizers and potential disease-modifying drugs in cholinergic neurons, of relevance for therapy of AD and insulin-related brain diseases.

We also addressed the effect of NGF on the insulin pathway in normal and insulin resistant conditions in vitro. NGF is the most relevant trophic and survival factor for cholinergic neurons [42, 43] and has been already shown to trans-activate the insulin pathway via TrkA-dependent signaling in PC12-derived neurons, thus suggesting a molecular link between NGF and insulin signaling in neurons [44]. Here, we show that NGF stimulates tyrosine phosphorylation of both IR and IRS_1_ in control cholinergic neurons (Fig. 2b-d), while in insulin resistant conditions NGF directly activates IRS_1_ (Fig. 3). Further, NGF restores IRS kinases activation level in insulin resistant cholinergic neurons (Fig. 3f-h). In both control (Fig. 2) and insulin resistant cholinergic neurons (Fig. 3), NGF is able to activate AKT (Figs. 2b, e and 3a, e; respectively) and increase c-Fos expression (Figs. 2f-g and 3i-j; respectively) and glucose uptake (Figs. 2h-i and 3k-l; respectively). Thus, we conclude that IRS_1_ is a key molecule of the insulin pathway responsible for glucose homeostasis and neuronal metabolism in cholinergic neurons. Moreover, NGF is able to stimulate the insulin pathway in control conditions and to improve insulin resistance induced by chronic high insulin in cultured cholinergic neurons.

There is general consensus on the cross-talk between insulin and Insulin like Growth Factor 1 (IGF1) pathways, resulting in common IRS-mediated PI3K/Akt and Ras/Raf/MAPK signaling initiated by hybrid receptors. Of note, IGF1 has been shown to promote the cholinergic phenotype of septal neurons and deregulation of IGF1 pathway has been implicated in neurodegenerative diseases [19, 20, 27]. Interestingly, our data indicate that NGF may counteract the detrimental effects of brain insulin resistance by acting on the IRS_1_ stimulation, the early, main and common effector of insulin and IGF1 receptors signalings. Thus, the involvement of IGF1 deserves future investigations and it is reasonable to hypothesize the co-occurrence of insulin and IGF1 resistances in our chronic high insulin model and a beneficial effect of NGF on IGF1 resistance in AD.

Furthermore, we deeper characterized the glucose metabolism addressing the involvement of the glucose transport protein (Glut) in these events. In particular, we focused on Glut2, a glucose sensor and Glut4, the prototypical insulin dependent Glut in peripheral organs [53]. While Glut2 is insulin-independent in the peripheral organs, its role in the CNS is not well understood. Indeed, Glut2 mRNA and protein have been reported within specific brain nuclei included the rodent and human basal forebrain [52, 65, 66], and have been implicated in neurotrophic control of glucose homeostasis in the rodent CNS [52, 67], as well as in zebrafish brain development [49]. Here, we observed that Glut2 massively translocates to the plasma membrane in the cell body, axon and dendrites in cholinergic neurons upon NGF treatment, and to a minor extent, following insulin stimulation (Figs. 2j and 4g).

As opposite, neuronal Glut4 localization is unaffected by insulin and NGF treatments (Suppl. Fig. 1e), neither by the IRS inhibitor NT157 (Suppl. Fig. 2i). These findings indicate that NGF- and insulin-mediated glucose uptake is mainly under Glut2 control in cultured cholinergic neurons (see also Fig. 2j), and suggest a minor role for Glut4 in our experimental conditions. In line with our observations, insulin failure in stimulating Glut4-dependent glucose uptake has been also reported in the human brain [20], supporting the idea that glucose metabolism regulation by Gluts is tissue and cell type specific.

Based on our observation that NGF mainly ameliorates insulin resistance by IRS_1_ activation, we thus asked whether the effect of NGF on glucose metabolism is mediated by IRS_1_ in cholinergic neurons. For this purpose, we used the specific IRS inhibitor NT157. Following IRS inhibition by NT157 in both control and insulin resistant conditions, NGF is not able anymore to stimulate AKT phosphorylation (Fig. 4a-b, respectively) and c-Fos expression (Fig. 4c-d, respectively), nor to induce plasma membrane re-localization of the glucose sensor Glut2 (Fig. 4g). Of note, NT157 treatment brought glucose uptake below insulin resistance level (Fig. 4e-f, respectively), pinpointing the essential role of IRS in Glut-mediated glucose uptake [10]. Moreover, the NGF system is known to induce c-Fos expression in the medial septum, resulting in elevated glucose uptake and c-Fos expression in vivo [48], while NGF withdrawal induces a repressed metabolic state inhibiting neuronal activity [67]. In line with these findings, our data extend previous observations pinpointing that NGF acts via IRS_1_ to promote downstream insulin pathway, glucose uptake and c-Fos expression in cholinergic neurons.

Further, we hypothesized that NGF induction of IRS_1_ relies on a direct interaction between IRS_1_ and the neurotrophin receptor TrkA. In fact, the NGF receptor TrkA and IRS_1_ can directly interact, as shown with the two hybrid system in yeast and induce downstream genes, like c-Fos [68]. In order to investigate whether TrkA and IRS_1_ endogenous molecules directly interact, we resorted to the PLA and found that NGF treatment does stimulate the binding of its receptor TrkA to IRS_1_ (Fig. 4k-m). Interaction between TrkA and IRS_1_ is even more pronounced after tyrosine phosphorylation of IRS_1_ (Fig. 4h-j), which is promoted by NGF (Fig. 2b, d). On the other hand, the IRS inhibitor NT157 completely abolished NGF effect on TrkA-IRS_1_ complex formation, suggesting the requirement of an active IRS_1_ for the TrkA-IRS_1_ interaction occurring under NGF stimulation.

Finally, we investigated the in vivo effect of NGF on the cholinergic system by nasal delivery to 3xTg-AD mice. Indeed, our findings indicate that NGF improves insulin resistance in the septum and induces the insulin pathway through tyrosine phosphorylation of IRS_1_, leading to the AKT-mediated pro-survival signaling. Noteworthy, NGF sustained the level of ChAT, a cholinergic marker affected by insulin resistance, possibly by ChAT stabilization, exerting further neuroprotective action for BFCN in AD-like neurodegeneration.

## Conclusion

Overall, these data indicate that NGF may elicit IRS_1_ stimulation in cholinergic neurons by-passing IR activation through IRS binding to the NGF receptor TrkA, as already demonstrated with other insulin sensitizers [56]. NGF-driven re-activation of the insulin pathway through disinhibition and tyrosine phosphorylation of IRS_1_, a primary gatekeeper in insulin signaling, is a potential novel strategy to slow cognitive decline in AD and diabetes-related brain insulin resistance. Ongoing collaborative studies are aimed at testing the in vivo potential of intranasal NGF administration on neuronal insulin resistance in AD and T2D mouse models. Given the growing demand for safe and effective disease-modifying drugs in AD, the design of novel NGF-based approach during the AD prodromal phase is of foremost clinical importance for future AD therapeutic strategies.

## Electronic supplementary material

Suppl. Fig. 1 **Medial septum responsivity to insulin: in vivo time course of insulin stimulation and effect of insulin and NGF on Glut4 in cholinergic neurons**. **(a-d)** Representative western blots (WB) are shown of pIR ^Y1150/1151^ (a-b), pIRS_1_^Y 608^ (a,c), and pAKT (a,d) on septal extracts from wild-type mice nasal administered with vehicle (veh) or insulin (INS, 0.125 IU) and sacrificed after 20′ and 40′. The results are reported as a percentage of the vehicle treated mice (veh). As shown, the levels of pIR^Y1150/1151^ (178.9 ± 7.2% of CTR, *p < 0.05; Suppl. Fig. 1b), pIRS_1_^Y608^ (158.13 ± 32.7, % of CTR, **p < 0.01; Suppl. Fig. 1c), and pAKT (169.6 ± 7.5% of CTR, **p < 0.01; Suppl. Fig. 1d) are increased 20′ after nasal insulin treatment (INS, 20′), and are back to control levels after 40′ (INS, 40′; 173.2 ± 38.9% of CTR; 131.8 ± 9.1% of CTR; 141.7 ± 7.7% of CTR; **p < 0.01). **(e)** Glut4 immunofluorescence staining and confocal imaging of cholinergic neurons treated with insulin (INS, 10 nM; 30′) or NGF (NGF, 100 ng/ml, 30′) demonstrate that neither insulin not NGF are able to induce translocation of Glut4 to the plasma membrane in cholinergic neurons in these experimental conditions. Scale bars: e = 50 μM. (PDF 1.34 MB)

Suppl. Fig. 2 **NT157 effect on cholinergic neurons survival and metabolism**. **(a)** Graph reporting IRS_1_ activation under insulin with or without the IRS inhibitor NT157 in cholinergic neurons. Insulin (INS, 251.1 ± 0.17% of CTR; ****p < 0.0001) is able to elicit IRS_1_ tyrosine phosphorylation. Preincubation with NT157 (10 μM; 2 h) completely abolishes IRS_1_ activation following INS (INS + NT157, 86.41 ± 0.06% of CTR; ****p < 0.0001), downsizing it to levels lower than in control neurons (**p < 0.01)**. (b)** Graph reporting pAKT activation under insulin with or without the IRS inhibitor NT157 in cholinergic neurons. Insulin (INS, 303.94 ± 12.81% of CTR; ***p < 0.001) is able to elicit IRS_1_ tyrosine phosphorylation. Preincubation with NT157 (10 μM; 2 h) completely abolishes IRS_1_ activation following INS stimulation, as expected (INS + NT157, 143.76 ± 2.91% of CTR; ***p < 0.001)**. (c)** Graph reporting the MTT assay of cholinergic neurons incubated with NGF alone (NGF, 100 ng/ml, 30′), or with NGF after preincubation with NT157 (10 μM; 2 h) (NGF + NT157), and showing that neuronal survival is not affected (*p* = 0.16) neither by NGF alone (100.4 ± 6.04% of CTR) nor by NGF after NT157 preincubation (117.09 ± 2.59% of CTR). **(d)** Representative WB of pIRS_1_^608^, total IRS_1_, Trk and β-actin in cholinergic neurons treated with NGF with and without the specific IRS inhibitor. **(e-f)** The graph illustrates the optical density analysis of the WB for IRS_1_ (e) and TrkA (f), showing that IRS_1_ (*p* = 0.9) and Trk (*p* = 0.56) levels are unchanged in NGF (96.37 ± 15.13% of CTR and 79.25 ± 9.73% of CTR, respectively) and in NGF + NT157 (91.53 ± 16.61% of CTR and 81.38 ± 22.6% of CTR, respectively) treated neurons. The results are reported as percentage of control cholinergic neurons (CTR, DIV10). **(g-h)** Representative WB of pAKT, AKT and β-actin upon treatment with insulin (INS, 10 nM, 30′), NGF (NGF, 100 ng/ml, 30′) or NGF after the incubation with the IRS inhibitor (NGF + NT157; 10 μM, 2 h) in control (g) and chronic high insulin (h) conditions. **(i)** Glut4 immunofluorescence staining and confocal imaging of cholinergic neurons treated with NGF alone (NGF, 100 ng/ml, 30′), or after preincubation with NT157 (10 μM; 2 h) (NGF + NT157) demonstrate that Glut4 do not translocate to the plasma membrane after NGF stimulation and it is not affected following IRS inhibition. Scale bars: *i* = 50 μM. (PDF 1.57 MB)

Suppl. Fig. 3 **TrkA/pIRS**_**1**_ **and TrkA/IRS**_**1**_ **immunolabelings in cholinergic neurons treated with NGF with or without NT157 preincubation (a-b)**. High magnification confocal images of TrkA and pIRS_1_^Y608^ (a) and TrkA and IRS_1_ (b) immunolabelings in cholinergic neurons (DIV10) treated with NGF with and without preincubation with NT157. Neurons were treated as indicated, were fixed, permeabilized, blocked with 5% BSA and incubated with (Suppl. Fig. 3a) mouse Trk, (*red*) and rabbit anti- pIRS_1_ (*green*) or with (Suppl. Fig. 3b) mouse Trk, (*red*) and rabbit anti-IRS_1_ (*green*) antibodies, overnight at 4 °C and then with an AlexaFluor-543 donkey anti-mouse and AlexaFluor-488 donkey anti-rabbit respectively, for 1 h. The panel reports the maximal projections of the z-stacks planes. (a) The pIRS_1_ (*green*) and TrkA (*red*) stainings are widespread distributed in control neurons (CTR), and TrkA is detectable also at the plasmamembrane, as expected. After NGF treatment (NGF) the signal mainly accumulated in the cell body. Following preincubation with NT157 (NGF + NT157), the NGF effect on pIRS_1_ is lost and the relative fluorescence signal is back to control levels. (b) IRS_1_ (*green*) and TrkA (*red*) stainings are mainly localized in the cytosol and can be found at both the cell body and dendrites of control neurons (CTR). Scale bars: a-b = 25 μM. (PDF 3.97 MB)

Suppl. Fig. 4 **PLA probes background signal is undetectable**. Untreated cholinergic neurons (DIV10) were fixed with PFA, blocked with normal donkey serum (10%, 1 h, RT). PLA assay was performed by omission of the primary antibodies and incubating neurons with PLA probes (anti-mouse-minus and anti-rabbit-plus secondary antibodies), as negative control for the PLA detection system. The PLA probes background signal was almost undetectable. Nuclei were counterstained with DAPI. Scale bar: 50 μM. (PDF 953 KB)

## Abbreviations

BFCN: Basal forebrain cholinergic neurons
AD: Alzheimer’s disease
NGF: Nerve growth factor
BFCS: Basal forebrain cholinergic system
MCI: Mild cognitive impairment
IR: Insulin receptors
IRS: Insulin receptor substrates
T2D: Type 2 diabetes
3xTg-AD: Triple transgenic AD
ChAT: Choline acetyltransferase
PLA: In situ proximity ligation assay
