# Supplementary figures and images for: The Medial Septum Is Insulin Resistant in the AD Presymptomatic Phase: Rescue by Nerve Growth Factor-Driven IRS1 Activation
Source: Mol Neurobiol. 2018 May 7;56(1):535–52. doi: 10.1007/s12035-018-1038-4 (PMC6334735; doi:10.1007/s12035-018-1038-4)

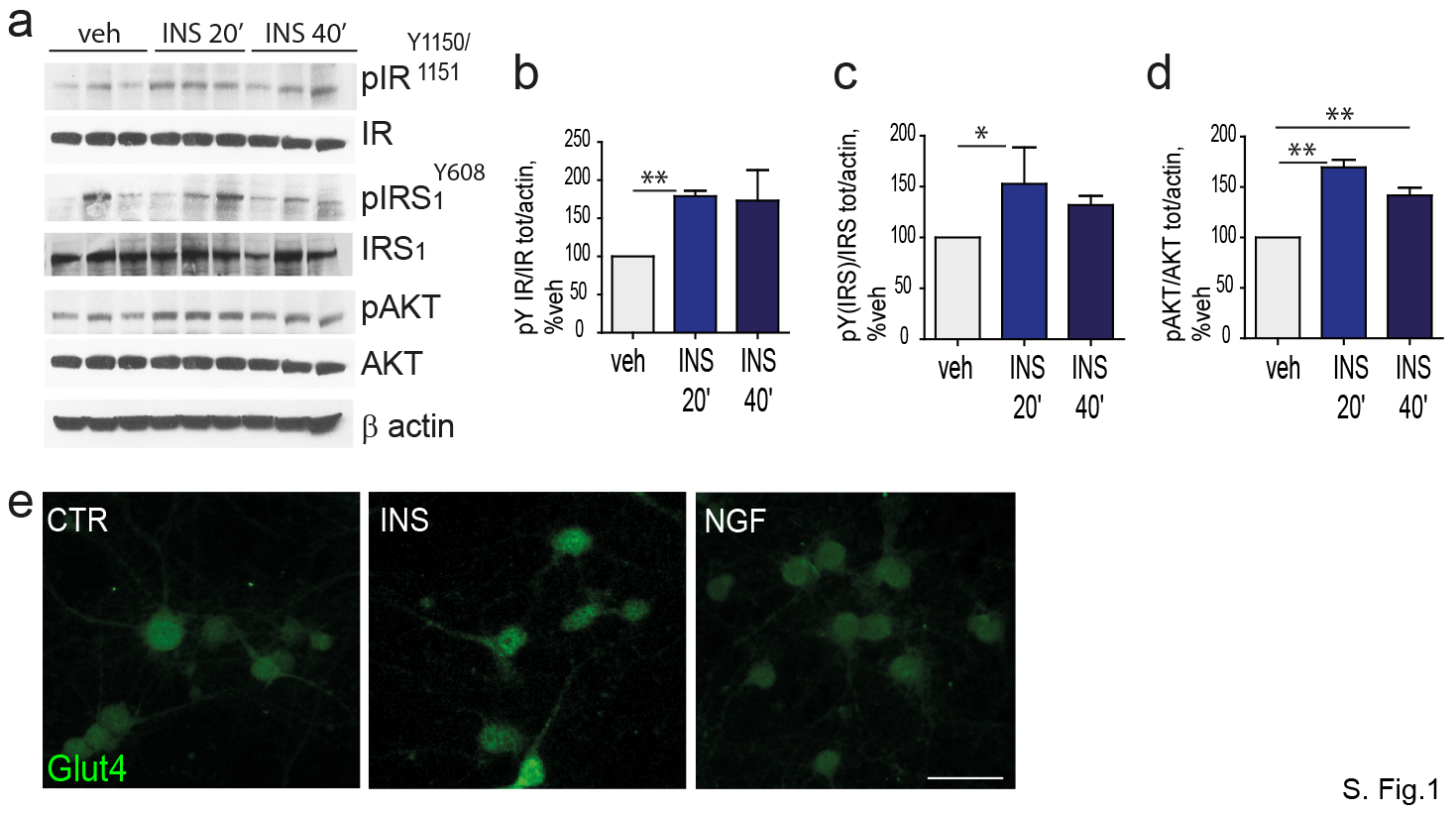

Supplement: Supplementary file 1 — Medial septum responsivity to insulin: in vivo time course of insulin stimulation and effect of insulin and NGF on Glut4 in cholinergic neurons. (a-d) Representative western blots (WB) are shown of pIR Y1150/1151 (a-b), pIRS1Y 608 (a,c), and pAKT (a,d) on septal extracts from wild-type mice nasal administered with vehicle (veh) or insulin (INS, 0.125 IU) and sacrificed after 20′ and 40′. The results are reported as a percentage of the vehicle treated mice (veh). As shown, the levels of pIRY1150/1151 (178.9 ± 7.2% of CTR, *p < 0.05; Suppl. Fig. 1b), pIRS1Y608 (158.13 ± 32.7, % of CTR, **p < 0.01; Suppl. Fig. 1c), and pAKT (169.6 ± 7.5% of CTR, **p < 0.01; Suppl. Fig. 1d) are increased 20′ after nasal insulin treatment (INS, 20′), and are back to control levels after 40′ (INS, 40′; 173.2 ± 38.9% of CTR; 131.8 ± 9.1% of CTR; 141.7 ± 7.7% of CTR; **p < 0.01). (e) Glut4 immunofluorescence staining and confocal imaging of cholinergic neurons treated with insulin (INS, 10 nM; 30′) or NGF (NGF, 100 ng/ml, 30′) demonstrate that neither insulin not NGF are able to induce translocation of Glut4 to the plasma membrane in cholinergic neurons in these experimental conditions. Scale bars: e = 50 μM. (PDF 1.34 MB) [file 12035_2018_1038_MOESM1_ESM.tif]

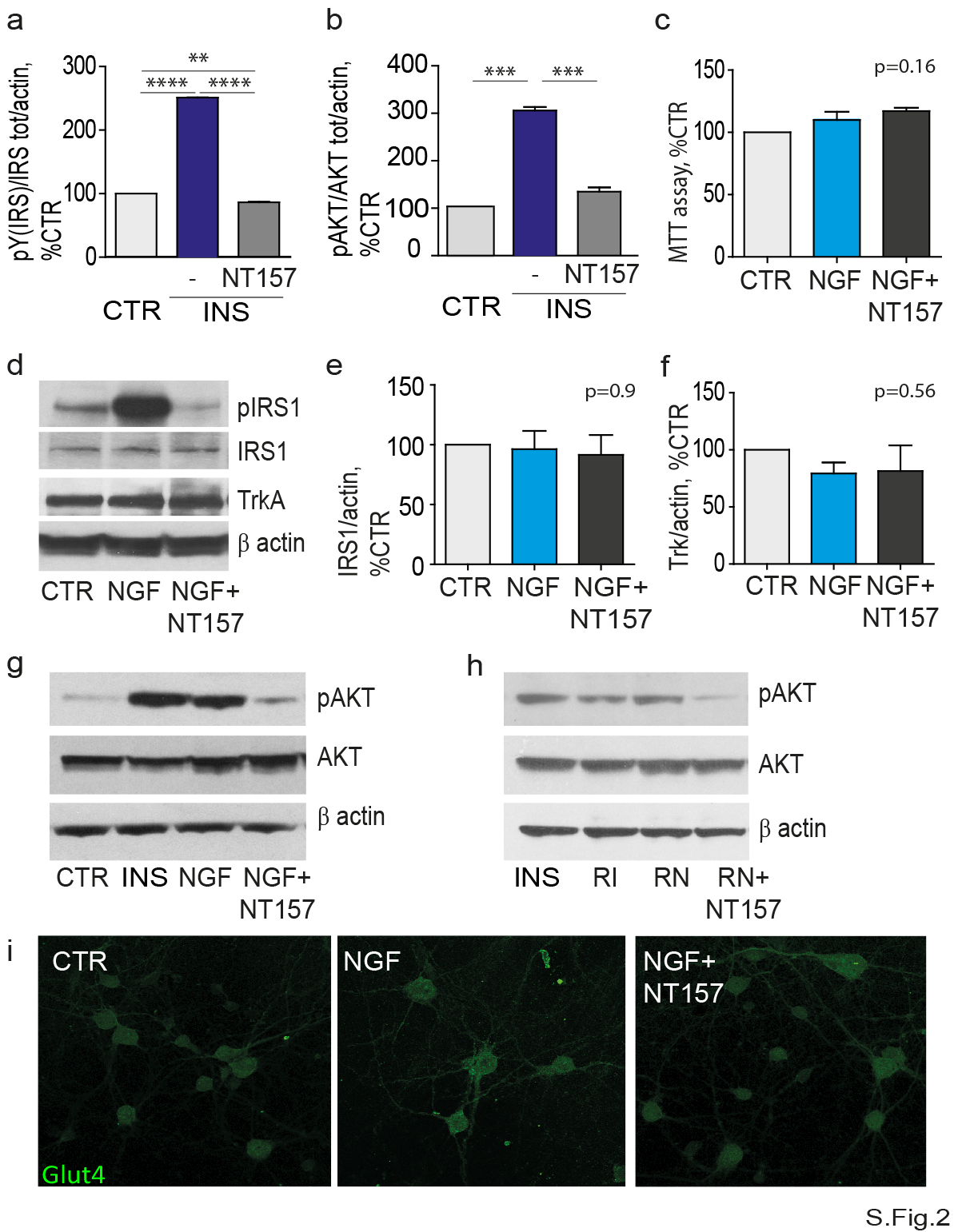

Supplement: Supplementary file 2 — NT157 effect on cholinergic neurons survival and metabolism. (a) Graph reporting IRS1 activation under insulin with or without the IRS inhibitor NT157 in cholinergic neurons. Insulin (INS, 251.1 ± 0.17% of CTR; ****p < 0.0001) is able to elicit IRS1 tyrosine phosphorylation. Preincubation with NT157 (10 μM; 2 h) completely abolishes IRS1 activation following INS (INS + NT157, 86.41 ± 0.06% of CTR; ****p < 0.0001), downsizing it to levels lower than in control neurons (**p < 0.01). (b) Graph reporting pAKT activation under insulin with or without the IRS inhibitor NT157 in cholinergic neurons. Insulin (INS, 303.94 ± 12.81% of CTR; ***p < 0.001) is able to elicit IRS1 tyrosine phosphorylation. Preincubation with NT157 (10 μM; 2 h) completely abolishes IRS1 activation following INS stimulation, as expected (INS + NT157, 143.76 ± 2.91% of CTR; ***p < 0.001). (c) Graph reporting the MTT assay of cholinergic neurons incubated with NGF alone (NGF, 100 ng/ml, 30′), or with NGF after preincubation with NT157 (10 μM; 2 h) (NGF + NT157), and showing that neuronal survival is not affected (p = 0.16) neither by NGF alone (100.4 ± 6.04% of CTR) nor by NGF after NT157 preincubation (117.09 ± 2.59% of CTR). (d) Representative WB of pIRS1608, total IRS1, Trk and β-actin in cholinergic neurons treated with NGF with and without the specific IRS inhibitor. (e-f) The graph illustrates the optical density analysis of the WB for IRS1 (e) and TrkA (f), showing that IRS1 (p = 0.9) and Trk (p = 0.56) levels are unchanged in NGF (96.37 ± 15.13% of CTR and 79.25 ± 9.73% of CTR, respectively) and in NGF + NT157 (91.53 ± 16.61% of CTR and 81.38 ± 22.6% of CTR, respectively) treated neurons. The results are reported as percentage of control cholinergic neurons (CTR, DIV10). (g-h) Representative WB of pAKT, AKT and β-actin upon treatment with insulin (INS, 10 nM, 30′), NGF (NGF, 100 ng/ml, 30′) or NGF after the incubation with the IRS inhibitor (NGF + NT157; 10 μM, 2 h) in control (g) and chronic [file 12035_2018_1038_MOESM2_ESM.tif]

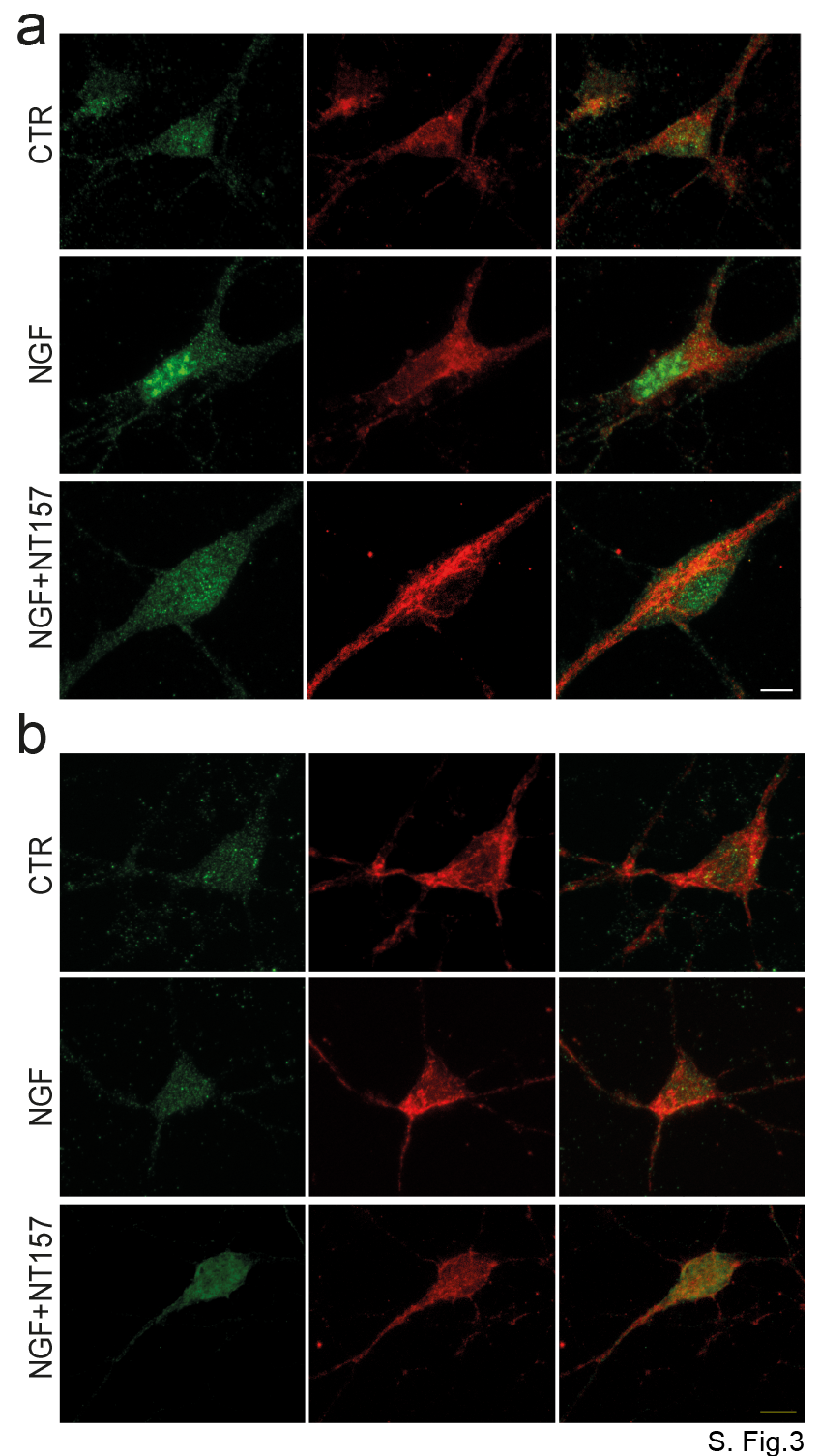

Supplement: Supplementary file 3 — TrkA/pIRS1 and TrkA/IRS1 immunolabelings in cholinergic neurons treated with NGF with or without NT157 preincubation (a-b). High magnification confocal images of TrkA and pIRS1Y608 (a) and TrkA and IRS1 (b) immunolabelings in cholinergic neurons (DIV10) treated with NGF with and without preincubation with NT157. Neurons were treated as indicated, were fixed, permeabilized, blocked with 5% BSA and incubated with (Suppl. Fig. 3a) mouse Trk, (red) and rabbit anti- pIRS1 (green) or with (Suppl. Fig. 3b) mouse Trk, (red) and rabbit anti-IRS1 (green) antibodies, overnight at 4 °C and then with an AlexaFluor-543 donkey anti-mouse and AlexaFluor-488 donkey anti-rabbit respectively, for 1 h. The panel reports the maximal projections of the z-stacks planes. (a) The pIRS1 (green) and TrkA (red) stainings are widespread distributed in control neurons (CTR), and TrkA is detectable also at the plasmamembrane, as expected. After NGF treatment (NGF) the signal mainly accumulated in the cell body. Following preincubation with NT157 (NGF + NT157), the NGF effect on pIRS1 is lost and the relative fluorescence signal is back to control levels. (b) IRS1 (green) and TrkA (red) stainings are mainly localized in the cytosol and can be found at both the cell body and dendrites of control neurons (CTR). Scale bars: a-b = 25 μM. (PDF 3.97 MB) [file 12035_2018_1038_MOESM3_ESM.tif]

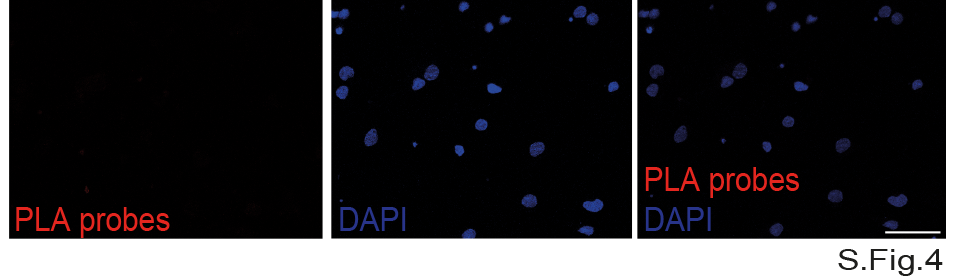

Supplement: Supplementary file 4 — PLA probes background signal is undetectable. Untreated cholinergic neurons (DIV10) were fixed with PFA, blocked with normal donkey serum (10%, 1 h, RT). PLA assay was performed by omission of the primary antibodies and incubating neurons with PLA probes (anti-mouse-minus and anti-rabbit-plus secondary antibodies), as negative control for the PLA detection system. The PLA probes background signal was almost undetectable. Nuclei were counterstained with DAPI. Scale bar: 50 μM. (PDF 953 KB) [file 12035_2018_1038_MOESM4_ESM.tif]
